# Indels, structural variation, and recombination drive genomic diversity in *Plasmodium falciparum*

**DOI:** 10.1101/gr.203711.115

**Published:** 2016-09

**Authors:** Alistair Miles, Zamin Iqbal, Paul Vauterin, Richard Pearson, Susana Campino, Michel Theron, Kelda Gould, Daniel Mead, Eleanor Drury, John O'Brien, Valentin Ruano Rubio, Bronwyn MacInnis, Jonathan Mwangi, Upeka Samarakoon, Lisa Ranford-Cartwright, Michael Ferdig, Karen Hayton, Xin-zhuan Su, Thomas Wellems, Julian Rayner, Gil McVean, Dominic Kwiatkowski

**Affiliations:** 1 MRC Centre for Genomics and Global Health, University of Oxford, Oxford OX3 7BN, United Kingdom;; 2 Malaria Programme, Wellcome Trust Sanger Institute, Hinxton CB10 1SA, United Kingdom;; 3 Wellcome Trust Centre for Human Genetics, University of Oxford, Oxford OX3 7BN, United Kingdom;; 4 Bowdoin College, Brunswick, Maine 04011, USA;; 5 Broad Institute of Harvard and MIT, Cambridge, Massachusetts 02142, USA;; 6 Department of Biochemistry, Medical School, Mount Kenya University, 01000 Thika, Kenya;; 7 Institute of Infection, Immunity and Inflammation, College of Medical, Veterinary and Life Sciences, University of Glasgow, Glasgow G12 8QQ, United Kingdom;; 8 Eck Institute for Global Health, Department of Biological Sciences, University of Notre Dame, Notre Dame, Indiana 46556, USA;; 9 Laboratory of Malaria and Vector Research, National Institute of Allergy and Infectious Disease, National Institutes of Health, Bethesda, Maryland 20892-9806, USA;; 10 Department of Statistics, University of Oxford, Oxford OX1 3LB, United Kingdom

## Abstract

The malaria parasite *Plasmodium falciparum* has a great capacity for evolutionary adaptation to evade host immunity and develop drug resistance. Current understanding of parasite evolution is impeded by the fact that a large fraction of the genome is either highly repetitive or highly variable and thus difficult to analyze using short-read sequencing technologies. Here, we describe a resource of deep sequencing data on parents and progeny from genetic crosses, which has enabled us to perform the first genome-wide, integrated analysis of SNP, indel and complex polymorphisms, using Mendelian error rates as an indicator of genotypic accuracy. These data reveal that indels are exceptionally abundant, being more common than SNPs and thus the dominant mode of polymorphism within the core genome. We use the high density of SNP and indel markers to analyze patterns of meiotic recombination, confirming a high rate of crossover events and providing the first estimates for the rate of non-crossover events and the length of conversion tracts. We observe several instances of meiotic recombination within copy number variants associated with drug resistance, demonstrating a mechanism whereby fitness costs associated with resistance mutations could be compensated and greater phenotypic plasticity could be acquired.

Genome variation in the eukaryotic pathogen *Plasmodium falciparum* underpins both fundamental biology, such as the ability of the parasite to evade the human immune response, and clinical outcomes, through the evolution of antimalarial drug resistance. This is of particular concern with the recent spread of resistance to front-line therapies in Southeast Asia (Ashley et al. 2014). High-throughput sequencing is a proven technology for the study of genome variation in *P. falciparum* and has yielded insights into parasite population structure (Manske et al. 2012; Miotto et al. 2013), transmission dynamics (Daniels et al. 2015), multiplicity of infection (Nair et al. 2014), the generation of antigenic diversity (Claessens et al. 2014), and the genetic basis for artemisinin resistance (Ariey et al. 2014). Despite these recent advances, our current understanding of *P. falciparum* genome variation remains incomplete due to multiple factors that are challenging both for sequencing technologies and for statistical methods used for variant discovery and genotyping. The highly compact 23-Mbp genome has an extremely biased nucleotide composition, with 80.6% (A + T) content overall and ∼90% (A + T) in noncoding regions (Gardner et al. 2002). As a result, many regions of the parasite genome are highly repetitive, with short tandem repeats and other low complexity sequences unusually abundant in both coding and noncoding regions (Gardner et al. 2002; DePristo et al. 2006; Zilversmit et al. 2010; Muralidharan and Goldberg 2013). Another difficulty is that parasite genes encoding antigenic targets of the host immune system tend to exhibit very high levels of diversity, and alternate alleles can be highly diverged from the reference sequence. An extreme example is the multicopy *var* gene family encoding erythrocyte surface antigens which can diversify within the course of a single infection by nonallelic recombination (Freitas-Junior et al. 2000; Bopp et al. 2013; Claessens et al. 2014).

These factors have limited progress, and there are a number of current knowledge gaps. There has been no comprehensive survey of insertion/deletion (indel) variation in *P. falciparum*, although there is evidence that indels may be unusually abundant (Su and Wellems 1996; Jeffares et al. 2007; Tan et al. 2010; Haerty and Golding 2011). Little is known about variation in noncoding regions, which could have a significant impact on phenotype by regulating gene expression (Gonzales et al. 2008; Mok et al. 2014). Knowledge of complex variation, where haplotypes are highly diverged from the reference genome, is constrained to a few well-studied genes such as *msp1* (Roy et al. 2008). A critical step in overcoming these obstacles is to have a reliable, empirical indicator of genotyping error, which allows genotyping methods to be calibrated and compared. There are many potential sources of error in the process of high-throughput sequencing and variant calling (Robasky et al. 2014), and different analytical methods may have different strengths and weaknesses. A proven approach is to sequence multiple individuals belonging to a pedigree and identify genotype calls in violation of Mendelian inheritance. A small number of Mendelian inconsistencies are expected due to de novo mutation, but the observation of many inconsistencies is a strong indicator of genotyping error. Mendelian errors can thus be used to calibrate methods and filter data (Saunders et al. 2007).

Here, we report an analysis of *P. falciparum* genome variation in the parents and progeny of experimental genetic crosses. We have sequenced all three crosses that have been published to date (Walliker et al. 1987; Wellems et al. 1990; Hayton et al. 2008) involving the parental clones 3D7, HB3, Dd2, 7G8, and GB4, representing a range of genetic and phenotypic diversity (Su et al. 2007; Ranford-Cartwright and Mwangi 2012). Although only a limited number of crosses are currently available, typically more than 30 genetically distinct progeny clones can be obtained from a single cross. The large number of progeny provides a higher power to observe Mendelian errors than smaller pedigrees or trios and thus to identify variants which are spurious or where genotyping is unreliable. We use a combination of methods for variant discovery to build a map of genome variation within each cross, integrating single nucleotide polymorphisms (SNPs), indel and complex polymorphisms, and spanning both coding and noncoding regions of the genome.

We also address open questions regarding meiotic recombination in *P. falciparum*, a key biological process that generates and maintains genetic diversity in natural parasite populations and thus contributes to parasite evolution. Previous studies using these crosses have estimated crossover (CO) recombination rates (Walker-Jonah et al. 1992; Su et al. 1999; Jiang et al. 2011) and provided evidence that non-crossover (NCO) recombination occurs (Su et al. 1999; Samarakoon et al. 2011a). Previous studies have also demonstrated that some recombination events occur within coding regions (Kerr et al. 1994) and suggested that recombination events are not uniformly distributed over the genome (Jiang et al. 2011). Here, we combine SNP and indel markers to obtain a resolution of ∼300 bp within each cross, which is sufficient to estimate rates for both CO and NCO recombination events and to study conversion tract lengths. This resolution is also sufficient to resolve the location of most recombination events relative to gene and exon boundaries and study the rate of intragenic recombination. We also investigate recombination in the context of two large regions of copy number amplification, both of which segregate in the crosses and are associated with antimalarial drug resistance (Wellems et al. 1990; Samarakoon et al. 2011b).

## Results

### Whole-genome sequencing and genome accessibility

Whole genomes of parent and progeny clones from the crosses 3D7 × HB3 (Walliker et al. 1987), HB3 × Dd2 (Wellems et al. 1990, 1991), and 7G8 × GB4 (Hayton et al. 2008) were sequenced using Illumina high-throughput technology (paired end, read length 75–100 bp depending on sample, insert size ∼100–200 bp) with the majority of samples obtaining an average depth above 100× (Table 1; Supplemental Table S1). All DNA libraries were derived from haploid parasite clones in culture, and sufficient DNA was available to use PCR-free library preparation throughout, which has been shown to reduce some of the biases associated with the AT-rich *P. falciparum* genome and hence improve the evenness of coverage across both coding and noncoding regions (Kozarewa et al. 2009). PCR can also induce false indels, providing an additional motivation for using PCR-free libraries (Fang et al. 2014). The clone HB3 is a parent in two crosses; however, because DNA samples were obtained from different sources and had different culturing histories, the two HB3 clones were sequenced and analyzed separately and are here labeled HB3(1) and HB3(2) for crosses 3D7 × HB3 and HB3 × Dd2, respectively. Biological replicates were obtained for several progeny clones, where libraries were created from DNA extracted from different cultures of the same parasite clone. These were also sequenced and genotyped separately to enable analysis of concordance between replicates.

**Table 1.**
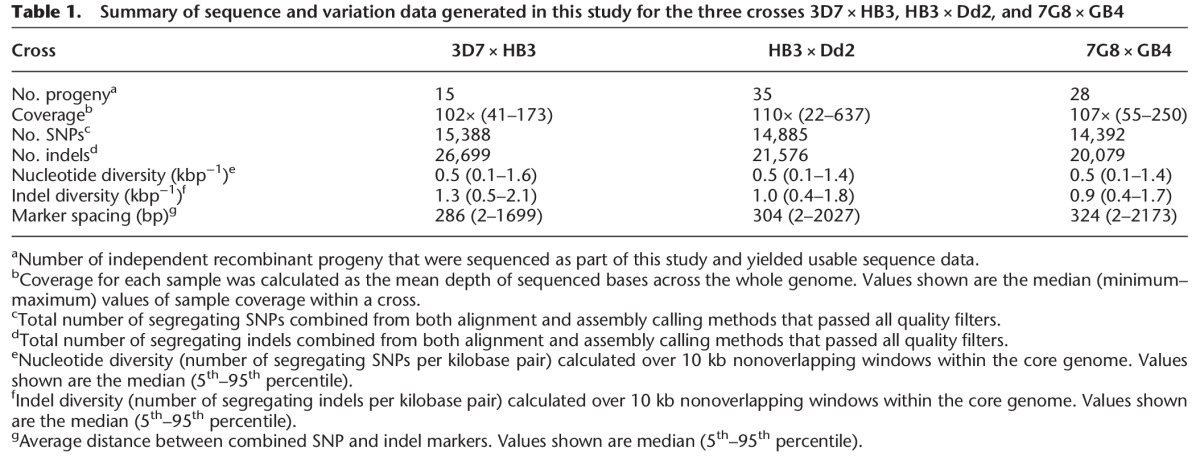
Summary of sequence and variation data generated in this study for the three crosses 3D7 × HB3, HB3 × Dd2, and 7G8 × GB4

Sequence reads from all samples were aligned to the 3D7 reference genome, and various metrics were calculated per genome position, including depth of coverage and averaging mapping quality. Visual examination of these data revealed a clear, qualitative difference between a core genome (20.8 Mb; 90%) comprising regions of near-complete coverage and unambiguous alignments in all samples; hypervariable regions (1.9 Mb; 8%) where accessibility was severely affected by both extensive paralogy and extreme divergence from the reference genome; and subtelomeric repeat regions (0.6 Mb; 2%) where accessibility was limited by repetitive sequence (Supplemental Figs. S1–S3; Supplemental Table S2). Hypervariable regions contained all genes in the *var* family, which are known to undergo frequent nonallelic recombination during mitosis (Bopp et al. 2013; Claessens et al. 2014), and almost all genes in the associated *rif* and *stevor* families. Hypervariable regions also corresponded closely with regions of heterochromatin (Flueck et al. 2009), confirming a strong association between chromatin state and qualitative differences in genome variability. All samples exhibited some degree of bias such that coverage was lower where (A + T) content was above 80%; however, the high depth of sequencing meant that >99.6% of the core genome was covered in all parental clones. Because of the poor accessibility of hypervariable and subtelomeric repeat regions, we excluded them from further study and limit ourselves to the core genome for the remainder of this paper.

### SNPs, indels, and complex variation within the core genome

SNPs, small indels, and regions of complex polymorphism were discovered and genotyped within each cross by two independent methods, one based on alignment of sequence reads to the 3D7 reference genome (Li and Durbin 2009; DePristo et al. 2011), the other based on partial assembly of sequence reads and comparison of assembled contigs (Iqbal et al. 2012). Methods for variant calling and filtering are given in Supplemental Methods. Variants where genotype calls in one or more progeny clones were inconsistent with Mendelian segregation (Mendelian errors) were used to calibrate variant filtering for both calling methods (Methods; Supplemental Figs. S4–S7). After variants were filtered, both methods achieved near-perfect concordance between biological replicates for both SNPs and indels (Supplemental Table S3), with, at most, 34/27,422 (0.12%) discordant genotypes from the alignment method and 18/33,801 (0.05%) from the assembly method for any single clone, demonstrating that the process from DNA extraction through sequencing and variant calling was highly reproducible. The inheritance of parental alleles within the progeny of each cross was also highly concordant when comparing SNPs with indels (>99.7% in all crosses) (Supplemental Fig. S8) or comparing results of the two variant calling methods (>99.8% in all crosses) (Supplemental Figs. S6, S7). To provide the greatest possible resolution for the present study, filtered variants called by each method were combined into a single call set for each cross (Table 1; Methods; Supplemental Information).

To estimate rates of false discovery and sensitivity, we compared variant alleles called in each of the HB3 replicates with the HB3 draft assembly (http://www.ncbi.nlm.nih.gov/nuccore/AANS00000000) and publicly available gene sequences for clone HB3 derived from Sanger sequencing (Supplemental Information; Supplemental Table S4). Although neither of these published resources have been independently validated, they provide the only pre-existing data on both SNP and indel polymorphism with which to compare. In comparisons at 32 genes, between 0.6%–2.7% of SNPs called by the alignment method and 0.0%–1.1% of SNPs called by the assembly method were not present in either the draft HB3 assembly or the HB3 Sanger sequences, providing an estimate for the false discovery rate (FDR) (Supplemental Table S5). Indel FDR estimates were higher for both calling methods, in the range 8.3%–12.5%; however, we noted a high rate of indel discordances between previously published sequences (Supplemental Fig. S17), suggesting the indel error rates in these sequences may be high, making reliable FDR estimation difficult. For sample HB3(1), we estimated SNP sensitivity above 84% and indel sensitivity above 70% for both calling methods; however, sensitivity was lower for HB3(2), particularly for the assembly calling method (Supplemental Table S5). This lower sensitivity for HB3(2) was partly due to a technical limitation of the assembly calling method, which was only capable of genotyping variants with a single nonreference allele (Supplemental Information).

### Indels are the most abundant form of polymorphism

**Figure 1.**
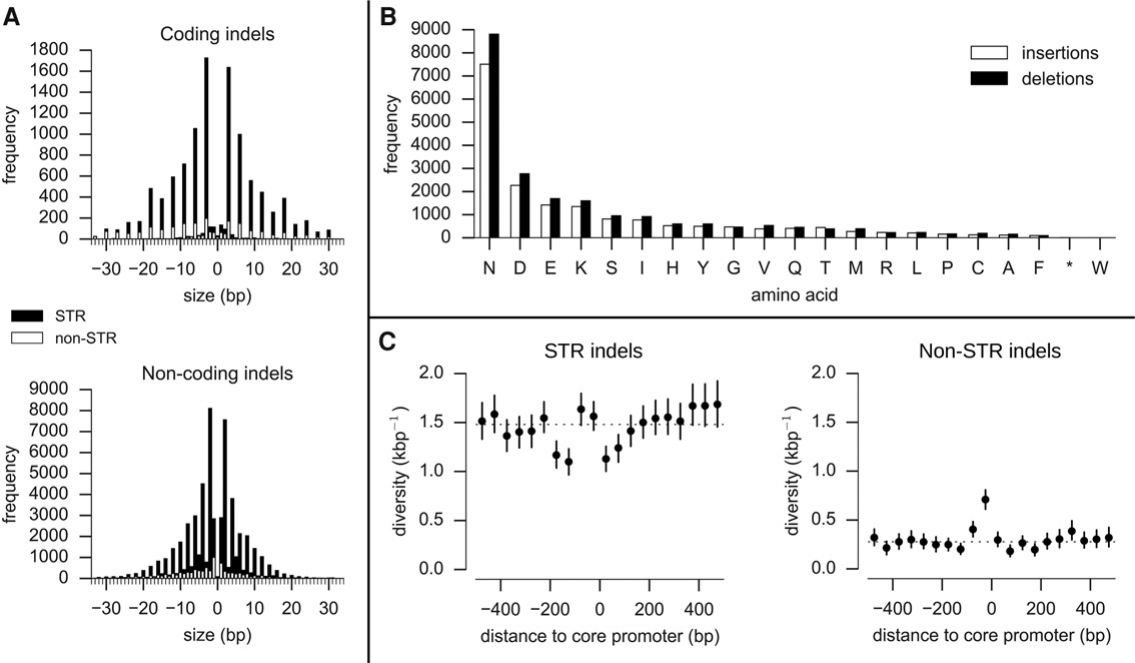
Properties of indels. (*A*) Indel size distribution (size > 0 are insertions, size < 0 are deletions). Solid black bars represent the frequency of indels that are expansions or contractions of short tandem repeats (STR); solid white bars represent the frequency of non-STR indels. Most coding indels are size multiples of 3, preserving the reading frame. Most noncoding indels are size multiples of 2, reflecting the abundance of poly(AT) repeats in noncoding regions. (*B*) Amino acids inserted and deleted (relative to the 3D7 reference genome). (*C*) Indel diversity in intergenic regions relative to the position of core promoters predicted by Brick et al. (2008). Each point represents the mean indel diversity in a 50-bp window at a given distance from the center of a core promoter. Vertical bars represent the 95% confidence interval from 1000 bootstraps. The dashed line is at the mean intergenic diversity for the given indel class (STR/non-STR).

Within the core genome, segregating indels were more abundant than SNPs in all three crosses (Table 1). Overall, 83% of indels were found in noncoding regions, where indels where three times more abundant than SNPs. Indels were also relatively abundant in coding regions, with the ratio of SNPs to indels being approximately 2:1. This relative abundance of indels is exceptionally high when compared with other species—for example, the SNP to indel ratio is approximately 10:1 in primates and 20:1 in bacteria (Chen et al. 2009). The vast majority of indels were expansions or contractions of short tandem repeats (STRs), i.e., microsatellites (Fig. 1A). In noncoding regions, 83% of indels were STR length variants, of which 71% were variants within poly(AT) repeats. In coding regions, 77% of indels were STR variants, of which the majority were within poly(asparagine) tracts (Fig. 1B). Longer repeat tracts were more polymorphic, and for any given tract length, longer repeat units were more stable (Supplemental Fig. S18), similar to indels in humans (Montgomery et al. 2013). Tandem repeat sequences are prone to slipped strand mispairing during DNA replication (Li et al. 2002; Lovett 2004) and are known to be associated with high rates of indel mutation (Montgomery et al. 2013). STRs are very common in noncoding regions of the *P. falciparum* core genome (Gardner et al. 2002), accounting for 34% of noncoding nucleotides. STRs are also unusually abundant in the exome (Tan et al. 2010; Muralidharan and Goldberg 2013), accounting for 11% of coding nucleotides. Hence, the high indel to SNP ratio may be accounted for by the abundance of STRs in the genome, coupled with the high mutability of STRs due to replication slippage.

Frame shift mutations within coding regions are expected to have severe consequences and hence be negatively selected. We found that 94% of coding indels were size multiples of three and hence preserved the reading frame, whereas most noncoding indels were size multiples of two, reflecting the abundance of poly(AT) repeats (Fig. 1A). Within noncoding regions, the phenotypic consequences of indel mutations are harder to predict. Relatively little is known about the transcription machinery in *P. falciparum*; however, Brick et al. (2008) predicted the location of core promoters upstream of genes based on a training set of known transcription start sites. We found that intergenic indel diversity displayed a specific architecture relative to the central positions of these predicted promoters, with an excess of non-STR indels within the first 50 bp upstream of the promoter center, and a deficit of STR indels 100–200 bp upstream of and 0–100 bp downstream from the promoter center (Fig. 1C). Indels in promoter regions have been shown to alter gene expression in other species (Li et al. 2002; Gymrek et al. 2015), but this remains to be verified in *P. falciparum*.

### Low nucleotide diversity is punctuated by complex variation in merozoite-stage genes

**Figure 2.**
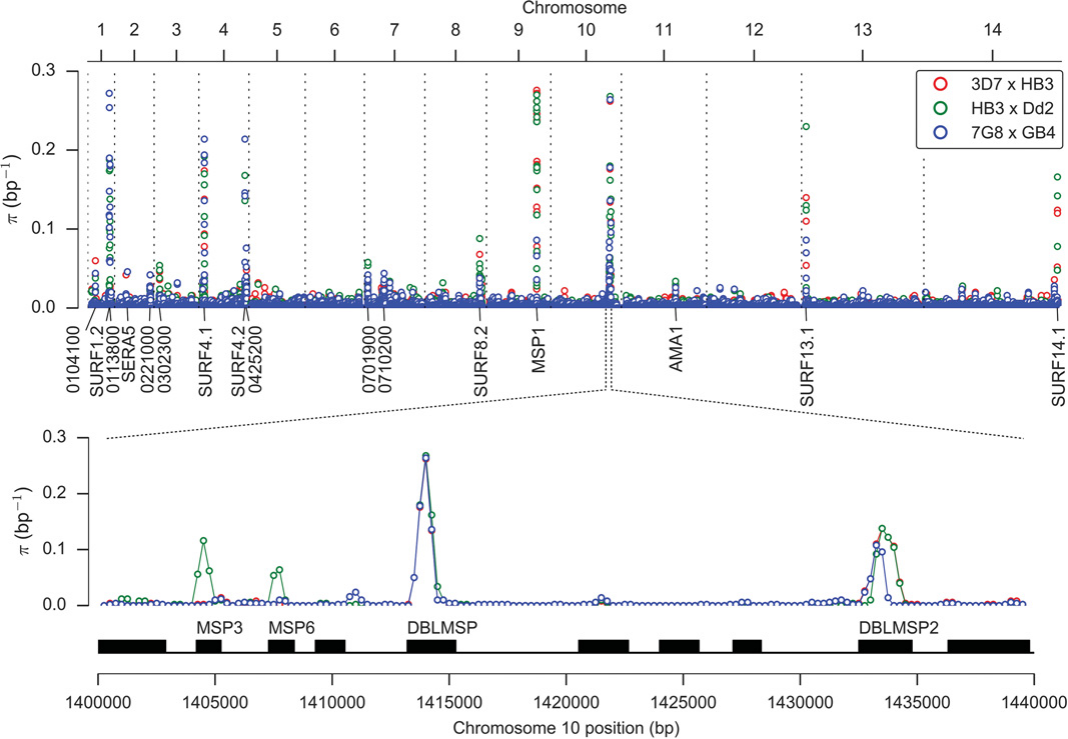
Variation in nucleotide diversity over the core genome. Nucleotide diversity is shown for each cross in 500-bp half-overlapping windows across the core genome (which excludes hypervariable regions containing *var*, *rif*, or *stevor* genes) using SNPs combined from both variant calling methods and passing all quality filters. The peak of nucleotide diversity on Chromosome 10 is expanded to show four distinct peaks due to genes encoding merozoite surface antigens MSP3, MSP6, DBLMSP, and DBLMSP2. All labeled loci (with the exception of AMA1) are sites of complex variation where assembly of sequence reads is required to determine the nonreference alleles.

Average nucleotide diversity across the core genome was 5 × 10^−4^ per bp in all three crosses (Table 1). However, this relatively low diversity was punctuated by 19 loci with highly diverged alleles, where local diversity over a region up to 2 kb was up to three orders of magnitude greater (Fig. 2). These divergent loci were found almost exclusively within coding regions of genes associated with the merozoite life cycle stage, where the parasite is briefly exposed to the host immune system before invading another erythrocyte, and include several well-studied merozoite surface antigens. The most extreme example was MSP1, a highly expressed protein located at the merozoite surface, where several regions of the gene are known to exhibit deep allelic dimorphism (Ferreira et al. 2003). The complex variation at these loci could not be accessed by the alignment method because sequences were too diverged from the reference genome, and hence coverage was locally patchy or nonexistent (Supplemental Fig. S9). However, the assembly method was able to construct complete and correct sequences for all parents and progeny in the two main divergent regions of MSP1 (blocks 4–11 and blocks 13–16 [Ferreira et al. 2003]) as verified by comparison with publicly available capillary sequence data. Other divergent genes where alleles could be assembled include four members of the *msp3* family (*msp3*, *msp6*, *dblmsp*, *dblmsp2*), six members of the *surf* family (*surf1.2*, *surf4.1*, *surf4.2*, *surf8.2*, *surf13.1*, *surf14.1*), and PF3D7_0113800 (encoding a DBL-containing protein with unknown function on Chromosome 1). A notable exception to the pattern of merozoite expression was PF3D7_0104100, which is transcribed by the sporozoite specifically within the mosquito salivary gland, suggesting involvement in the early stages of infection (Lasonder et al. 2008). Several of these genes are vaccine candidates and/or are being actively studied for their role in erythrocyte invasion, and comprehensive knowledge of variation at these loci is essential for the design of effective vaccines and invasion assays.

### Meiotic crossover and non-crossover recombination

These crosses are currently the only available experimental system for *P. falciparum* where meiotic recombination can be directly observed. For each cross, SNP and indel variants combined from both calling methods were used as a set of segregating markers for analyses of meiotic recombination (Table 1). The average distance between markers was ∼300 bp in all three crosses, at least an order of magnitude greater resolution than available previously (Jiang et al. 2011). In eukaryotes, double-strand breaks (DSBs) initiated during meiosis are resolved by either crossover or non-crossover between homologous chromosomes (Hastings 1992; Baudat and de Massy 2007; Mancera et al. 2008; Youds and Boulton 2011). A CO is a reciprocal exchange accompanied by a conversion tract, whereas an NCO is a conversion tract without reciprocal exchange (also known as a gene conversion, although NCO events can occur in either coding or noncoding regions) (see also Fig. 1 in Youds and Boulton 2011). We inferred recombination events from the size and arrangement of parental haplotype blocks transmitted to the progeny (Methods; Supplemental Information) yielding a total of 1194 COs, 230 NCOs, and 331 conversion tracts for further analysis (Supplemental Figs. S10, S11).

### Gene coding regions are warm-spots and centromeres are cold-spots of CO recombination

**Figure 3.**
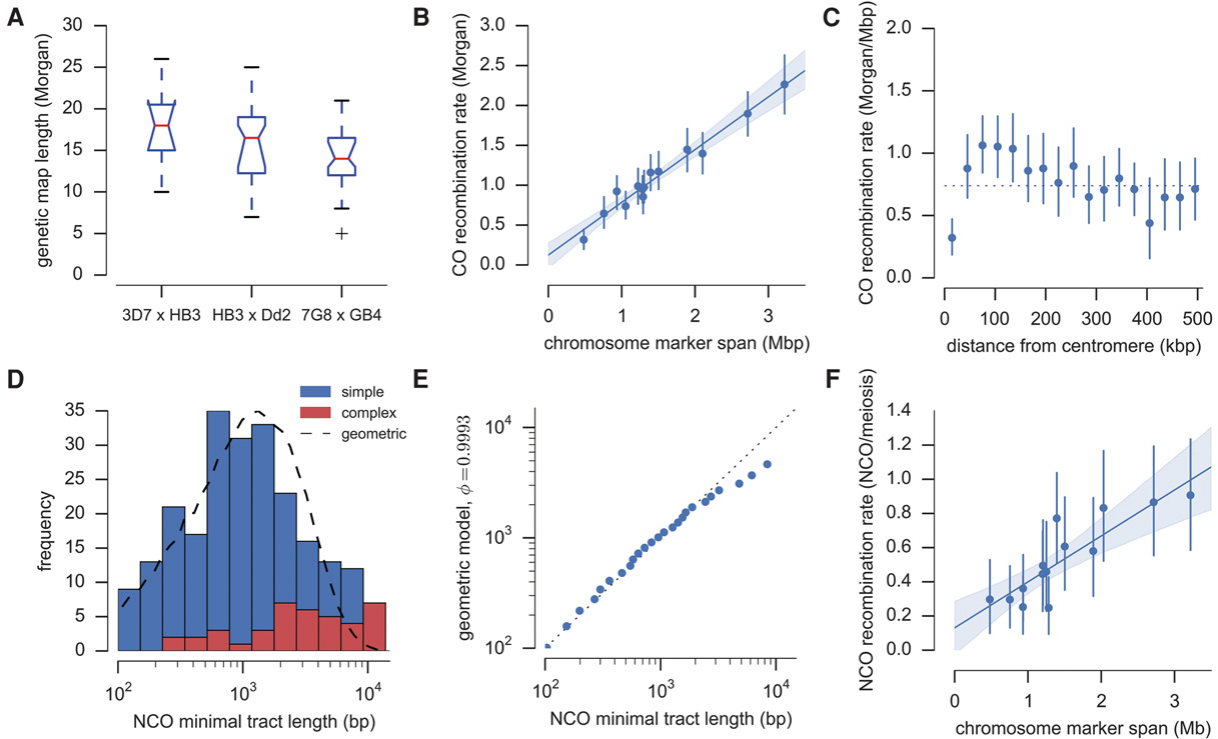
Crossover (CO) and non-crossover (NCO) recombination parameters. (*A*) Genetic map length by cross. For each cross, the red line shows the median map length averaged over progeny; boxes extend from lower to upper quartiles. (*B*) Map length by chromosome. Each point shows the mean map length for a single chromosome averaged over progeny, with an error bar showing the 95% confidence interval from 1000 bootstraps. The line shows a fitted linear regression model with shading showing the 95% bootstrap confidence interval. (*C*) CO recombination rate relative to centromere position as given by the genome annotation. Error bars show the 95% confidence interval from 1000 bootstraps. (*D*) NCO tract length distribution. The dashed line shows the distribution of minimal tract lengths that would be observed with the available markers if NCO tract lengths follow a geometric distribution with parameter φ= 0.9993. (*E*) Quantile-quantile plot of actual NCO minimal tract lengths versus the expected distribution of minimal tract lengths that would be observed with the given markers if NCO tract length is modeled as a geometric distribution with parameter φ = 0.9993. The data fit the model well except for an excess of tracts with minimal length greater than ∼3 kb. (*F*) NCO frequency by chromosome, adjusted for incomplete discovery of NCO events. Error bars and linear regression as in *B*.

Combining CO events from all three crosses, the total map length of the core genome was 15.7 Morgans (95% confidence interval: 14.8–16.6). The total marker span of the physical chromosomes was 21.16 Mb, giving an average CO recombination rate of 13.5 kb/cM (95% confidence interval: 12.7–14.3). The map length varied between crosses, with 3D7 × HB3 highest (17.7 Morgans), HB3 × Dd2 intermediate (16.0 Morgans), and 7G8 × GB4 lowest (14.3 Morgans), although this difference was marginally significant (*P* = 0.06, Kruskal-Wallis H-test) (Fig. 3A). There was a strong linear correlation between chromosome size and map length, with 0.55 Morgan predicted for the smallest chromosome (Fig. 3B), consistent with ∼0.5 Morgan expected if crossovers play an essential role in chromosome segregation, and thus the recombination rate is calibrated to produce at least one CO per bivalent (Baudat and de Massy 2007; Mancera et al. 2008; Martinez-Perez and Colaiácovo 2009). The centromeres were cold-spots of CO recombination, as expected from studies in other eukaryotes and confirming previous data from the 7G8 × GB4 cross (Jiang et al. 2011), although the effect was highly localized (Fig. 3C). Within ∼30 kb of the centromere, the CO rate was significantly lower; however, between ∼80–120 kb from the centromere, the rate was slightly higher than average.

Due to the high marker density, in many cases it was possible to resolve the location of CO events relative to individual gene and exon boundaries. Of the 1194 CO events, 396 (33%) were observed within a gene (intragenic COs), 162 (13%) were within an inter-genic region, and 636 (53%) were ambiguous (flanking markers spanned a gene boundary). The number of intragenic CO events was significantly higher than expected if CO events were distributed uniformly over the genome (*P* = 0.001 by Monte Carlo simulation). Of the 396 intragenic COs observed, 298 (75%) were observed within an exon, three (1%) were within an intron, and 95 (24%) localized across an exon boundary. The number of COs observed within exons was also significantly higher than expected if COs occurred uniformly within genes (*P* < 0.001 by Monte Carlo simulation). Thus, a substantial fraction of all CO events occurred within coding regions, in contrast with humans where the majority of recombination occurs within hotspots that preferentially occur near genes but outside of the transcribed domain (Myers et al. 2005).

Jiang et al. (2011) previously analyzed recombination in the 7G8 × GB4 cross using a tiling array, finding evidence that the CO recombination rate was not uniform over the genome. To test the hypothesis of fine-scale variation in the recombination rate, we combined the COs from all three crosses and fitted a Poisson regression model to counts of CO events in nonoverlapping windows over the core genome. The model was fitted against 17 parameters, including distance to centromere, number of coding bases, number of tandem repeat bases, number of SNPs, number of indels, and distance to various sequence motifs. We analyzed different window sizes from 50 kbp down to 2 kbp, in each case performing a one-tailed test for overdispersion (Cameron and Trivedi 1990) to assess whether there remained any variation in recombination rate not explained by the model. At the coarser scale of 50-kb windows, the number of coding bases (*P* = 3.6 × 10^−10^) and the number of tandem repeat bases (*P* = 6.5 × 10^−5^) within a window were positive predictors of CO count. At the finer scale of 5-kb windows, the number of coding bases (*P* = 1.5 × 10^−11^), number of tandem repeat bases (*P* = 3.0 × 10^−10^), distance to centromere (*P* = 0.0001), and distance to subtelomere (*P* = 0.0004) were all positive predictors of CO count. At 2-kb windows, these four predictors remained significant and, in addition, the number of indels (*P* = 7.6 × 10^−6^) and distance to the degenerate triplet repeat motif [TG]GA[TA]GAAG[AG][TG]GA (Jiang et al. [2011], motif C) (*P* = 0.0005) were negative predictors. No overdispersion was found for the model fitted against 50-kb windows (*P* = 0.51); however, at finer scales, significant overdispersion was detected (*P* < 0.001), suggesting there may be other sequence motifs or parameters associated with fine-scale variation in CO recombination rate that remain to be discovered.

### Estimation of conversion tract length and NCO recombination rate

Of the 331 conversion tracts observed, an outlying group of seven very long (>18 kb) complex tracts was found, described further below. Of the remaining 324 tracts, 94 were associated with a CO and 230 were assumed to be NCO conversion tracts. Fifty percent of observed NCO conversion tracts had a minimal size <1 kb and 73% were smaller than 2 kb (Fig. 3D). The relatively small size of conversion tracts and the available marker density means that some NCO events would not have been observed, because we required tracts to span at least two markers separated by >100 bp. To estimate the NCO recombination rate and true tract length distribution, the incomplete discovery of NCO events and bias toward discovery of longer tracts has to be taken into account. In *Drosophila*, the distribution of conversion tract lengths was found to fit a geometric model, with parameter φ determining the per-base-pair probability of extending a tract (Hilliker et al. 1994). We found that a geometric model also provided a good fit for the observed distribution of tract lengths in the present study, with φ = 0.9993 corresponding to a mean tract length of 1.4 kb, although there was a small excess of tracts observed with minimal length >3 kb (Fig. 3D,E). Assuming this model for the true tract length distribution, simulations predicted an NCO discovery rate of 40% for HB3 × Dd2, 39% for 7G8 × GB4, and 45% for 3D7 × HB3 where the marker density was slightly higher (Supplemental Information).

Adjusting for incomplete discovery, the average rate of NCO recombination over all three crosses was estimated at 7.5 NCO/meiosis (0.36 NCO/meiosis/Mb); thus, COs were roughly twice as common as NCOs. The 95% confidence interval for the NCO recombination rate based on sampling error was 6.8–8.1 NCO/meiosis; however, this does not account for additional uncertainty in the estimation of NCO discovery rates for each cross. As with CO events, there was a significant enrichment of NCO events within genes (*P* = 0.002 by Monte Carlo simulation), with 37 (16%) NCO conversion tracts falling entirely within a gene, 110 (48%) spanning a gene boundary, 35 (15%) entirely spanning a gene, and 14 (6%) intergenic.

As mentioned above, seven apparently long (>18 kb) complex conversion tracts were found. Two of these tracts occurred in clone JF6 (7G8 × GB4) within a 60-kb region on Chromosome 11 and thus appear to be part of a single complex long-range recombination event involving a total of 20 switches in inheritance (Supplemental Fig. S12). Two biological replicates of clone JF6 were sequenced and genotyped in this study, and the pattern of recombination was identical in both replicates. Similar observations were made in clones C04 (3D7 × HB3) and 3BD5 (HB3 × Dd2) (Supplemental Fig. S12). These observations do not fit well with conventional DSB repair pathways leading to normal CO and NCO events, suggesting other repair pathways may also be used during meiosis that have more radical results in terms of generating novel haplotypes (Mancera et al. 2008).

### Recombination within copy number variants spanning drug resistance genes

Clone Dd2 is known to have a threefold amplification spanning the multidrug resistance gene *mdr1*, conferring mefloquine resistance (Cowman et al. 1994). Amplifications have also been found spanning *gch1*, conferring resistance to anti-folate drugs, in both HB3 and Dd2, although the amplifications are different in size and extent (Kidgell et al. 2006; Heinberg et al. 2013). The *mdr1* amplification segregates in the progeny of HB3 × Dd2 (Wellems et al. 1990), and there is evidence that meiotic recombination has occurred within the amplified region in two progeny clones (Samarakoon et al. 2011a). The *gch1* amplifications have been shown to segregate in the progeny of HB3 × Dd2, although one progeny clone (CH3_61) appeared to inherit both parental amplifications superposed (Samarakoon et al. 2011a). Some form of recombination within the amplified region could explain this phenomenon, although the exact nature of the recombination is uncertain. All five parental clones have been shown to carry some form of amplification spanning *gch1* (Kidgell et al. 2006; Sepúlveda et al. 2013); thus, the sequence data generated in this study provide an opportunity to elaborate on previous results for HB3 × Dd2 and extend the analysis of copy number variant (CNV) transmission and recombination at drug resistance loci to 3D7 × HB3 and 7G8 × GB4.

**Figure 4.**
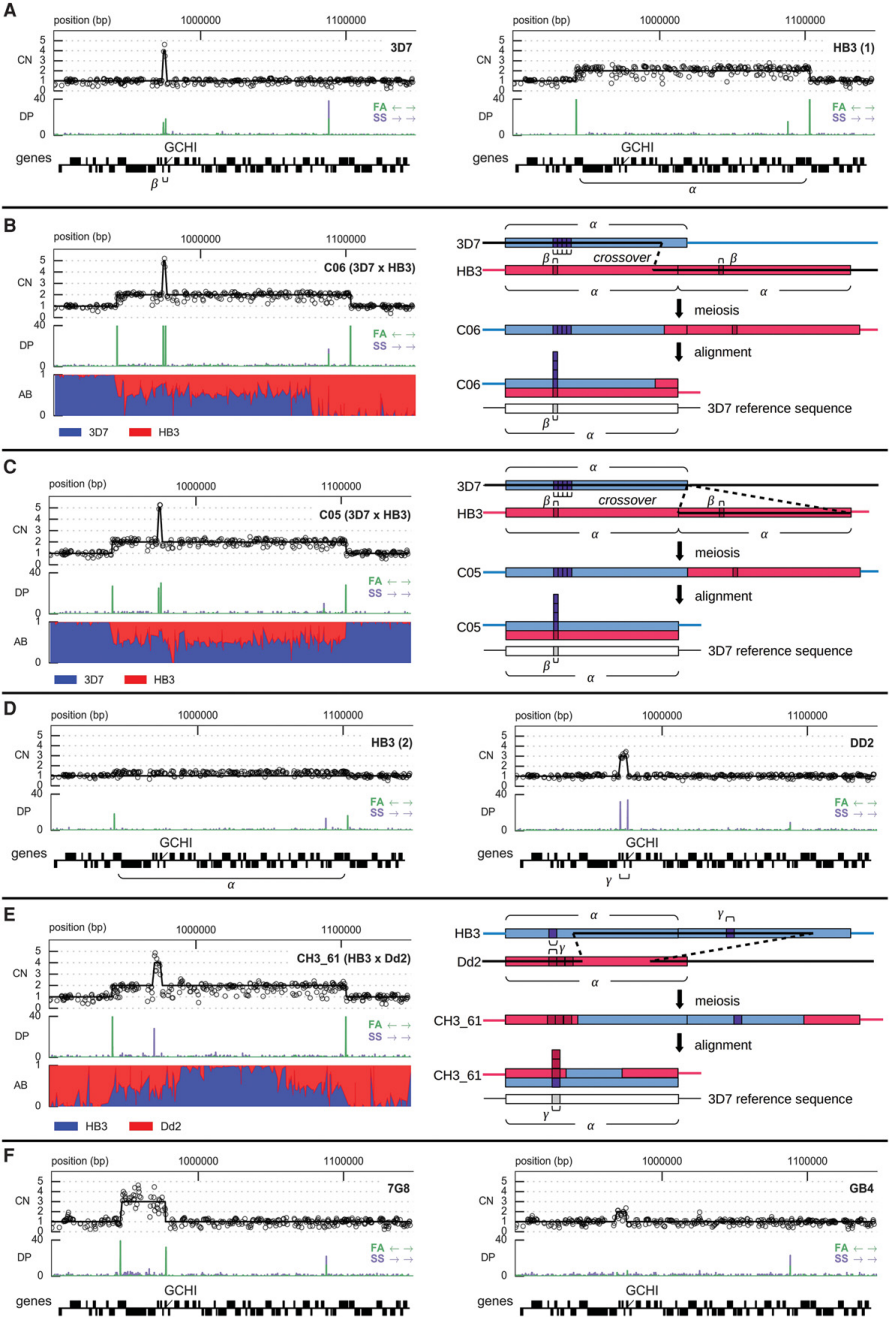
Copy number variation and recombination spanning the anti-folate resistance gene *gch1* on Chromosome 12. (*A*) CNVs in the 3D7 and HB3(1) parental clones; α labels the segment amplified in HB3, β labels the segment amplified in 3D7. (*B*) CNV and recombination in clone C06, progeny of 3D7 × HB3. AB = fraction of aligned reads containing the first parent's allele. (*C*) CNV and recombination in clone C05, progeny of 3D7 × HB3. AB = fraction of aligned reads containing the first parent's allele. (*D*) CNVs in the HB3(2) and Dd2 parental clones; γ labels the segment amplified in Dd2. Note that the HB3(2) clone sequenced here appears to be a mixture, with a minor proportion of parasites carrying the amplification visible in HB3(1). (*E*) CNV and recombination in clone CH3_61, progeny of HB3 × Dd2. AB = fraction of aligned reads containing the first parent's allele. (*F*) CNVs in the 7G8 and GB4 parental clones. CN = copy number; markers show normalized read counts within 300-bp nonoverlapping windows, excluding windows where GC content was below 20%; solid black line is the copy number predicted by fitting a Gaussian hidden Markov model to the coverage data (Supplemental Information). DP = depth of coverage (number of aligned reads), FA = reads aligned facing away from each other (expected at boundaries of a tandem array), SS = reads aligned in the same orientation (expected at boundaries of a tandem inversion).

We combined data on depth of sequence coverage and the orientation of aligned read pairs to study CNV alleles in all three crosses (Methods; Supplemental Information). The sequence data confirmed a threefold amplification in Dd2 spanning *mdr1* and transmission as either two or three copies to 14 progeny of HB3 × Dd2 (Supplemental Fig. S13). Evidence for amplifications spanning *gch1* was also clear in all parental clones (Fig. 4). The 3D7 reference sequence (version 3) has only a single copy of *gch1*; however, all CNV studies, including ours, have found the 3D7 clone to carry multiple copies of *gch1*, indicating an error in the reference sequence. Parental amplifications spanning *gch1* all differed in extent and copy number, confirming previous findings (Nair et al. 2008; Sepúlveda et al. 2013). The alignment of read pairs indicated that the Dd2 amplification was arranged as a tandem inversion (Fig. 4D), whereas 3D7, HB3, and 7G8 carried tandem arrays (Fig. 4A,E), adding further evidence for the independent origin of these CNV alleles. The HB3(2) sample appeared to be a mixture, with ∼20% of parasites retaining the duplication found in HB3(1) and 80% having no amplification (Fig. 4D), which is not unexpected given that amplifications can be lost in culture in the absence of drug pressure, leading to a mixed colony of parasites (Cowman et al. 1994). We found short (<50 bp) regions of homology at the putative breakpoints in 3D7, HB3, and 7G8, consistent with previous CNV studies in *P. falciparum* (Nair et al. 2007), suggesting these tandem amplifications arose via unequal crossing-over. Transmission of *gch1* CNV alleles was consistent with Mendelian segregation in the progeny of all three crosses except for two progeny of 3D7 × HB3 (C05, C06) and one progeny of HB3 × Dd2 (CH3_61), where both parental alleles appeared to be inherited together (Fig. 4; Supplemental Figs. S14–S16).

### Recombination within amplified regions leads to pseudo-heterozygosity

To further explore the apparent non-Mendelian inheritance of *gch1* CNV alleles, we considered possible recombination events that could explain the observed patterns of inheritance. Depending on how homologous chromosomes align during meiosis, a crossover within a region that is duplicated in one parent could result in a daughter that maintains the same duplication but inherits one copy from either parent for some portion of the amplified region. Within such a segment, a haploid individual would become effectively diploid heterozygous for any SNP, indel, or smaller CNV variants that segregated between the two parents. We used the alignment of sequence reads from each progeny clone to the 3D7 reference genome to look for evidence of pseudo-heterozygosity and thus recombination within amplified regions. At segregating sites within a region of pseudo-heterozygosity, reads supporting each parental allele should appear in a roughly 1:1 ratio, whereas elsewhere only one parental allele should be observed.

At the *gch1* locus, both clones C05 and C06 inherited the large 161-kb duplication from parent HB3 as well as the smaller 2-kb fourfold amplification from parent 3D7 spanning *gch1* only (Fig. 4). C06 had a region of heterozygosity spanning the leftmost 130 kb of the region duplicated in HB3 but was apparently homozygous for the remainder of this region (Fig. 4B). The most parsimonious explanation is that a single crossover occurred within the region duplicated in HB3. Clone C05 had a region of heterozygosity spanning the entire region duplicated in HB3, with borders that appeared to coincide closely with the breakpoints of the duplication (Fig. 4C). This is harder to explain as it would require at least two crossover events at or close to the borders of the duplicated region, which would seem improbable unless the CNV breakpoints are also prone to meiotic crossover (Völker et al. 2010). For both clones C05 and C06, *gch1* itself lay within the region of heterozygosity, thus one copy of *gch1* was inherited from HB3 and four copies from 3D7. At the same locus, clone CH3_61 inherited the 161-kb duplication from HB3 as well as the 5-kb threefold tandem inversion from Dd2 (Fig. 4E). Two separate regions of heterozygosity were visible at either end of the HB3 duplicated region, which can be explained if two crossover events occurred. Again, *gch1* was within the region of heterozygosity, and thus CH3_61 acquired one copy from HB3 and three copies from Dd2. We also confirmed previous evidence for recombination within the 82-kb amplification spanning *mdr1* in two progeny of HB3 × Dd2 (Samarakoon et al. 2011a). Clone QC23 had a region of heterozygosity spanning the leftmost 16 kb of the segment, and CH3_61 was heterozygous for the rightmost 40 kb spanning *mdr1* itself (data not shown). Both of these are consistent with a single crossover having occurred within the amplified region.

## Discussion

Genome variation has been studied in *P. falciparum* using a variety of technologies (e.g., Su and Wellems 1996; Kidgell et al. 2006; Mu et al. 2007; Neafsey et al. 2008; Manske et al. 2012). However, we have described the first integrated analysis of SNPs, indels, and complex polymorphisms, spanning both coding and noncoding regions of the core genome. Our analysis excluded hypervariable regions containing *var* genes, because divergence from the reference genome combined with paralogous sequence present severe challenges to both alignment- and assembly-based variant calling methods using short sequence reads. Longer sequence reads will be required to overcome these challenges and fully characterize the structural rearrangements between *var* genes that occur during mitosis (Claessens et al. 2014).

We found that indels were the most common polymorphism within the core genome. Indels were exceptionally common in noncoding regions and displayed a specific pattern of abundance relative to the position of predicted core promoters. Repeat length variants within regulatory regions have been found in other species and shown to affect gene activity (Li et al. 2002; Muzzey et al. 2013). Using the HB3 × Dd2 cross, Gonzales et al. (2008) showed that both *cis* and *trans* genetic variation influences gene expression in *P. falciparum*, including a major *trans* regulatory hotspot coinciding with the amplification spanning *mdr1*. Variation in gene regulation could affect clinically relevant phenotypes including drug sensitivity; e.g., Mok et al. (2014) found that deletion of a promoter upstream of *mrp2* altered sensitivity to quinoline drugs. The data on noncoding variation presented here could provide a starting point for further experimental work to explore the impact of noncoding variation in *P. falciparum*.

*P. falciparum* is a sexually reproducing eukaryotic pathogen, and these crosses provided the first demonstration that parasites undergo meiotic recombination while in the mosquito (Walliker et al. 1987). We combined data from all three crosses to estimate a CO recombination rate in the range 12.7–14.3 kb/cM, in close agreement with previous studies (Jiang et al. 2011). We also estimated that CO events are approximately twice as frequent as NCO events, similar to yeast (Mancera et al. 2008) but contrasting with humans (Padhukasahasram and Rannala 2013) and *Drosophila* (Miller et al. 2012), where NCOs may be more common than COs. Samarakoon et al. (2011b) studied two progeny of HB3 × Dd2 using 454 sequencing and observed a similar number of CO and putative NCO events in both progeny samples. It is not clear why our estimated NCO rate is lower, especially as marker resolution is an order of magnitude higher in this study, and thus power to observe NCO tracts should be higher. We found that conversion tract lengths in *P. falciparum* are comparable to yeast (Mancera et al. 2008) but longer than humans (Jeffreys and May 2004) and *Drosophila* (Hilliker et al. 1994; Miller et al. 2012). Our observations of apparent long-range complex recombination events spanning >60 kb in some progeny do not fit well with current models for eukaryotic recombination pathways and remain to be explained, although similar events have been observed in yeast (Mancera et al. 2008).

In many eukaryotes, the recombination rate is known to be variable over the genome, with most recombination concentrated within narrow hotspots (Myers et al. 2005; Drouaud et al. 2006; Tsai et al. 2010). Previous work on the 7G8 × GB4 cross suggested that the *P. falciparum* genome may also contain recombination hotspots (Jiang et al. 2011). At scales of 2–5 kb we found that, within the core genome, recombination rates were lower near centromeres and subtelomeres, and higher CO recombination rates were associated with repeat-rich coding sequence, including one of the hotspot motifs previously identified, a 12-bp degenerate triplet repeat (Jiang et al. 2011). Coding regions have higher (G + C) content than noncoding regions in *P. falciparum*, and so this could indicate a preference for double-strand break formation in regions with higher (G + C) content; however, this does not explain the bias toward repetitive sequence. At finer scales, the parameters we modeled did not fully explain the variation in recombination rates observed; thus, there may be other factors driving local variation in recombination rate. However, given the total number of CO events (1194) observed in this study, we cannot be confident about the existence of any specific recombination hotspots. For example, taking a simple definition of hotspot as any 5-kb window with two or more CO events in a single cross, only seven of 204 hotspots would be discovered in more than one cross and none in all three crosses. Further crosses combined with fine-scale recombination maps estimated from population data would help to resolve these questions.

We have extended the previous observation of a recombination event within the *gch1* amplification in the HB3 × Dd2 cross (Samarakoon et al. 2011a) to illustrate two other cases of meiotic recombination within amplifications at this locus. We have also shown that all of these events generate regions of pseudo-heterozygosity within a progeny clone where both parental sequences are inherited and maintained within a single haploid genome. Such events could have important evolutionary consequences. First, drug resistance mutations may confer a fitness cost relative to the wild-type allele in the absence of drug pressure (Anderson et al. 2009; Kondrashov 2012; Rosenthal 2013) and may also confer both resistance to one class of drugs and sensitivity to another (Anderson et al. 2009). The process of amplification followed by homologous recombination provides a mechanism by which both mutant and wild-type alleles can be acquired. If both alleles are expressed, this could produce a new codominant phenotype, compensating for the lower fitness of either allele alone. The acquisition of both alleles also creates an opportunity to epigenetically silence one allele and switch expression between alleles if conditions change. Epigenetic switching between duplicated genes has been shown to occur at the *clag3* locus, altering susceptibility to the antibiotic blasticidin S (Mira-Martínez et al. 2013). Over a longer timescale, gene duplication combined with recombination may facilitate functional diversification, enabling adaptation to different or novel conditions. For example, in plant viruses, gene duplication and recombination may have facilitated adaptation to a wide range of host species (Valli et al. 2007).

Finally, we remark on the connection between indel and CNV mutation. Previous studies have found that CNV breakpoints almost invariably occur at sites with some degree of local homology, suggesting that amplifications are due to improper pairing of homologous chromosomes followed by unequal crossover (Nair et al. 2007). Tandem repeats are highly abundant in the *P. falciparum* core genome; thus, there are many sites of ectopic homology providing opportunities for improper pairing during meiosis. Nair et al. (2007) also showed that CNV breakpoints are found in repeat region that are slightly longer than the genome-wide average; thus, variation in tandem repeat length could shift the amplification potential to a different set of loci. We have shown here that indel variants within tandem repeat regions are abundant throughout the core genome, and thus amplification potential is likely to be highly dynamic and variable within natural populations.

The core genome of *P. falciparum* thus appears stable yet poised to undergo rapid evolution within any region that comes under selection. This may become particularly relevant as malaria elimination intensifies in Southeast Asia, applying ever stronger selective pressures to parasite populations.

## Methods

### Whole-genome sequencing

All sequencing was carried out using Illumina high-throughput technology as described in Manske et al. (2012), except that the PCR-free method of library preparation as described in Kozarewa et al. (2009) was used.

### Variant calling

Variants were called by two independent methods. The alignment method followed GATK best practice recommendations (DePristo et al. 2011; Van der Auwera et al. 2013) with some adaptations for *P. falciparum*. The assembly method used Cortex (Iqbal et al. 2012) following the independent workflow. Mendelian errors were used to calibrate variant filtering methods. Filtered variants from both calling methods were then combined into a single set of segregating variation for each cross.

### Inference of CO and NCO recombination events and conversion tracts

The combined variant call sets were used to infer recombination events via the inheritance of parental haplotype blocks. The calling algorithm identified conversion tracts and called CO and NCO events from the size and arrangement of parental haplotype blocks within each progeny clone, based on the assumption that two CO events are unlikely to occur within close proximity, and therefore short haplotype blocks (minimal size < 10 kb) are due to conversion tracts.

### Recombination analyses

To calculate the map length for each cross, the identity map function was used because the marker density was high, and thus we assumed all crossovers were observed. To estimate the true conversion tract length distribution, the parameter φ (per-base-pair probability of extending a tract) was fitted via Monte Carlo simulations. These simulations also estimated the fraction of conversion tracts that would be discovered given the markers available in each cross. The rate of NCO recombination was then estimated by adjusting the number of observed NCO events by the estimated discovery rate.

To study variation in recombination rate over the genome, we fitted a Poisson regression model to counts of CO events in nonoverlapping windows over the genome using the glm() function in R (R Core Team 2015) and tested for overdispersion using the AER package (Kleiber and Zeileis 2008). The following parameters were included in the model: distance to centromere, distance to subtelomere, number of coding bases within the window, number of tandem repeat bases within the window, percent (G + C), number of segregating SNPs, number of segregating indels, nearest distance to each of the five motifs identified in Jiang et al. (2011), and nearest distance to the common repeat motifs poly(A), poly(T), poly(AT), and poly(AAT).

### Copy number variation

The genome was divided into 300-bp nonoverlapping bins, and the number of reads whose alignment started within each bin was calculated for each sample. Bins where the GC content was lower than 20% were excluded from coverage analyses due to coverage bias in most samples. The binned read counts were then normalized by dividing by the median read count found within the core regions of Chromosome 14. Copy number state was predicted in all samples by fitting a Gaussian hidden Markov model to the normalized coverage data.

## Data access

Genome sequence data from this study have been submitted to the European Nucleotide Archive (ENA; http://www.ebi.ac.uk/ena) under study accession numbers PRJEB2146 (3D7 × HB3) and PRJEB2136 (HB3 × Dd2 and 7G8 × GB4). Alignments of sequence reads to the 3D7 reference genome have been submitted to the ENA under study accession number PRJEB14481. A mapping from clone identifiers to ENA run accessions is given in Supplemental Table S1. All variant calls from this study have been submitted to the European Variation Archive (EVA; http://www.ebi.ac.uk/eva) under study accession number PRJEB14423. Data from this study can also be downloaded from a public FTP site at ftp://ngs.sanger.ac.uk/production/malaria/pf-crosses/ and can be explored interactively via a Web application at http://www.malariagen.net/apps/pf-crosses/ (Supplemental Methods section 1.5, Supplemental Fig. S19).

## Supplementary Material

Supplemental Material
