# Supplementary material for: Indels, structural variation, and recombination drive genomic diversity in Plasmodium falciparum
Source: Genome Res. 2016 Sep;26(9):1288–99. doi: 10.1101/gr.203711.115 (PMC5052046; doi:10.1101/gr.203711.115)
Supplement: Supplemental Material [file supp_26_9_1288__index.html]

Indels, structural variation, and recombination drive genomic diversity in Plasmodium falciparum — Supplemental Material 

# Indels, structural variation, and recombination drive genomic diversity in *Plasmodium falciparum*

## Supplemental Material

- Supplemental\_Information.pdf
