# Supplementary material for: Indels, structural variation, and recombination drive genomic diversity in Plasmodium falciparum
Source: Genome Res. 2016 Sep;26(9):1288–99. doi: 10.1101/gr.203711.115 (PMC5052046; doi:10.1101/gr.203711.115)
Supplement: Supplemental Material [file supp_gr.203711.115_Supplemental_Information.pdf]

Genome variation and meiotic recombination in *Plasmodium falciparum*: insights from deep sequencing of genetic crosses

Supplemental information

Table of contents

1. Supplemental methods.....2
  1.1. Whole genome sequencing.....2
  1.2. Sequence alignment and genome region classification.....2
  1.3. Variant discovery and genotype calling.....4
    1.3.1. Alignment-based calling method (BWA/GATK).....4
    1.3.2. Assembly-based calling method (Cortex).....6
    1.3.3. Combined callset.....7
    1.3.4. Genotype concordance between biological replicates.....7
    1.3.5. Estimation of FDR and sensitivity.....7
  1.4. Recombination analyses.....8
    1.4.1. Calling CO and NCO recombination events.....8
    1.4.2. Estimation of CO and NCO recombination parameters.....10
    1.4.3. Recombination within CNVs.....11
  1.5. A Web application to facilitate data exploration and re-use.....11
2. Supplemental tables.....13
3. Supplemental figures.....21
4. References.....41

All data described in this supplement can be accessed via the MalariaGEN website at http://www.malariagen.net/data/pf-crosses-1.0. A web application is also available at http://www.malariagen.net/apps/pf-crosses which provides interactive tools for visualising and exploring the data.

All code used to generate figures and analyses for the main paper and for supplementary information is contained in IPython notebooks in the GitHub repository at https://github.com/wtchg-kwiatkowski/pfx-paper-2015 and can be browsed via http://nbviewer.ipython.org/github/wtchg-kwiatkowski/pfx-paper-2015/tree/20141022/.

1. Supplemental methods

1.1. Whole genome sequencing

Sample preparation and sequencing was performed as described in (Manske, Miotto et al., 2012) except that PCR-free library preparation was used throughout (Kozarewa et al. 2009). All sequences were deposited in the European Nucleotide Archive and a mapping from sample identifiers to ENA accessions is given in **Table S1** and in the web application at http://www.malariagen.net/apps/pf-crosses/.

Typically in high throughput sequencing studies of humans or other higher eukaryotes multiple sequencing runs will be obtained for each sample, then data from each run (lane) are combined to increase coverage. However in this study a single sequencing run was sufficient to obtain ~100X coverage of the *P. falciparum* genome, so only a single sequencing run was obtained for each sample. Samples that represented biological replicates (DNA derived from the same clone but obtained from different cultures) were treated separately, with separate DNA library preparation and sequencing runs. Thus in this study there is always a one-to-one mapping from sample (biological replicate) to sequence run.

For convenience we use a three-part identifier for each sample, e.g., “3D7/PG0051-C/ERR019061”, where the first part identifies the clone (e.g., “3D7”), the second part is our internal lab identifier for the DNA sample (e.g., “PG0051-C”), and the third part is the accession for the sequencing run at the ENA (e.g., “ERR019061”). The second and third parts are redundant, because as mentioned above there is a one-to-one mapping from sample to sequencing run, however we include both for transparency. The data files available from the FTP site and the web application use the same identifier system for consistency.

1.2. Sequence alignment and genome region classification

Sequence reads from each sample were aligned to the 3D7 version 3 reference genome using BWA (Li and Durbin 2009) version 0.6.1-r104 with the following parameter settings:

```
bwa aln -n 0.01 -k 4
bwa sampe
```

We found that the custom parameters to the `aln` command served to slightly increase the sensitivity and improve consistency of the alignment in regions with clusters of SNPs, such as the polymorphisms found at the chloroquine resistance locus (Fidock et al. 2000), however the vast majority of alignments were identical under the custom and default settings (data not shown). Sequence alignments can be downloaded as BAM files from the FTP site at ftp://ngs.sanger.ac.uk/production/pf-crosses/1.0/ and browsed via the web application at http://www.malariagen.net/apps/pf-crosses/.

Various metrics were then calculated from the alignments of each sample. These metrics were computed per genome position based on the pileup of aligned reads, using the program `pysamstats`[1]. Metrics calculated include the total depth of coverage, percentage of reads aligned

1 https://github.com/alimanfoo/pysamstats

in a proper pair (i.e., in correct orientation and reasonable distance apart, as defined by the aligner), average mapping quality and percentage of reads aligned ambiguously (mapping quality zero).

Alignment metrics for each of the parental samples were then plotted for each chromosome, alongside other metrics derived from the reference genome sequence, including the %GC content in a 300bp window and the non-uniqueness score (defined as the smallest k-mer size at which all k-mers overlapping a given position are unique within the genome (Manske, Miotto et al., 2012); a high score for this metric is bad, in the sense that it indicates low uniqueness). An example plot for sample HB3/PG0052-C/ERR019054 and chromosome 4 is shown in **Figure S1**. The alignments themselves were also visualised using the LookSeq web application (Manske & Kwiatkowski, 2009).

From these visualisations a clear, qualitative distinction could be seen between regions of the genome with consistent coverage across all parent samples, and regions with significant alignment issues in one or more parents. To capture these large-scale qualitative differences we defined the following heuristic scheme for classifying genome regions:

- **Core –** Regions with near-continuous coverage in all samples, with a high percentage of reads mapping in a proper pair and a low proportion of reads aligned ambiguously.
- **Subtelomeric Hypervariable –** Gene-containing regions towards the sub-telomere of a chromosome, with patchy and/or highly variable coverage in one or more samples and/or a low proportion of reads aligned in a proper pair and/or a high percentage of ambiguous alignments and/or a high percentage of aligned bases mismatching the reference.
- **Internal Hypervariable –** Gene-containing regions towards the centromere of a chromosome, with patchy and/or highly variable coverage in one or more samples and/or a low proportion of reads aligned in a proper pair and/or a high percentage of ambiguous alignments and/or a high percentage of aligned bases mismatching the reference.
- **Subtelomeric Repeat –** Gene-free regions with repetitive sequence at the end of a chromosome, typically with highly variable coverage and a high percentage of ambiguous alignments.
- **Centromere –** Centromere as given in the GeneDB genome annotation.

Within each chromosome we defined boundaries for these regions by eye from the visualisations described above. **Table S2** gives the region boundaries, **Figure S2** shows a map of the genome regions defined, and **Figure S3** gives a summary of alignment statistics for each parental clone by region class. At least 99.6% of core genome positions were covered in all parents, and at least 98.8% of the core genome was covered by unambiguously mapped reads.

Our definition of the core genome is subjective, and more sophisticated methods could be devised to partition the genome into regions with different alignment characteristics. However the contrast between these different regions of the genome is very striking, and we believe the definitions given here capture the major qualitative features in a useful way.

The genome region classification can be browsed alongside coverage, mapping quality and other metrics via the web application URL http://www.malariagen.net/apps/pf-crosses/. A BED file

defining the region boundaries can be downloaded from the FTP site at
ftp://ngs.sanger.ac.uk/production/pf-crosses/1.0/.

1.3. Variant discovery and genotype calling

1.3.1. Alignment-based calling method (BWA/GATK)

The alignment-based calling method used the Genome Analysis Tool Kit version 2.6-4-g3e5ff60 (McKenna et al. 2010) and followed best practice recommendations as published at the time (DePristo et al. 2011; Van der Auwera et al. 2013).

Starting from the reads aligned to the 3D7 version 3 reference genome as described above, the following steps were performed to prepare the BAM files. Using Picard tools version 1.77 the commands CleanSam, FixMateInformation, AddOrReplaceReadGroups and MarkDuplicates were run on each BAM file in that order.

Base quality score recalibration (BQSR) was then applied to the BAM files. BQSR empirically recalibrates the base quality scores reported for each base in each sequence read, by observing the correlation between mismatches in the aligned sequence reads and various covariates, including the original base quality reported by the sequencing machine, in addition to other factors like the local sequence context. BQSR thus relies on the assumption that a substantial number of bases mismatching the reference in aligned sequence reads are due to sequencing error and not true variation, alignment error or some other type of artefact. From a visual inspection of the alignments for the parental clones (see, e.g., **Figure S1**) it was apparent that the mismatch rate within hypervariable regions was extremely high, and given the other alignment symptoms in hypervariable regions including patchy coverage and ambiguous mapping, we assumed the vast majority of these mismatches were due to divergence between clones and not sequencing error. To avoid hypervariable regions overwhelming BQSR we limited the building of the covariates table to the core genome. BQSR also requires a set of known variant positions to exclude when building the covariates table. To bootstrap BQSR we created an initial set of variant calls for each cross from the raw BAM files using UnifiedGenotyper, then filtered these calls to exclude any that had less than 2 confident (GQ = 99) ALT calls, contained Mendelian errors, had more than 2 missing calls or were part of a homopolymer run of length 5 or more.

We then applied INDEL realignment to the recalibrated BAMs. Each BAM file was realigned separately, but to improve the sensitivity of INDEL realignment we provided as input the set of bootstrap INDEL calls obtained from the previous BQSR step, which has the effect of sharing information about possible INDEL alleles between samples. All other settings were default.

We then generated a raw variant callset from the realigned BAMs using UnifiedGenotyper run under a haploid model (-ploidy 1).

The next step was to empirically recalibrate variant quality scores (VQSR). VQSR requires at least a positive training set of known true variants, and optionally one or more negative training sets of sites where variant calls are likely to be spurious. We defined a positive training set for each cross by selecting variants from the raw callset that segregated within the cross according to Mendelian inheritance (i.e., parents had different genotypes, progeny had no Mendelian errors) and also produced highly parsimonious patterns of inheritance (i.e., did not induce an unrealistically high

rate of recombination). Specifically, the positive training sets included only SNP and INDEL variants within the core genome, with no missing calls, no non-Mendelian calls, and no calls inducing an apparent double-crossover at a single variant. We also created two negative training sets for each cross, the first containing variants with Mendelian errors, the second containing variants inducing single-variant double-crossovers in one or more samples.

We then applied VQSR to each cross separately. VQSR was run for SNPs with the following options:

- -an QD -an DP -an MQ -an UQ -an HaplotypeScore -an ReadPosRankSum -an FS --target_titv 1.0 --percentBadVariants 0.1 --stdThreshold 10.0 --maxGaussians 6

VQSR for INDELs was run with the following options:

- -an QD -an DP -an MQ -an UQ -an HaplotypeScore --target_titv 1.0 --percentBadVariants 0 --stdThreshold 10.0 --maxGaussians 6

“UQ” is the non-uniqueness score define above and the other annotations are standard INFO annotations produced by GATK.

To verify that the VQSR runs had been effective we plotted the rate of Mendelian error against the number of variants for different thresholds of the VQSLOD score (similar to an ROC curve) (**Figure S4**). For all three crosses and for both SNPs and INDELs, we observed an inflection point in these curves, corresponding to a Mendelian error rate of approximately 0.05% or ~1 Mendelian error in 2000 genotype calls. Thresholds (minimum values) were chosen for the VQSLOD separately for SNPs and INDELs in each of the three crosses at the inflection point in the curve. For SNPs the thresholds were 3D7xHB3: 2.5, HB3xDd2: 3, 7G8xGB4: 4; for INDELs the thresholds were 3D7xHB3: 1, HB3xDd2: 1.5, 7G8xGB4: 1.8.

We generated a final, analysis-ready VCF for each cross by adding the following filter annotations:

- LOW_CONFIDENCE – Variant confidence is low (VQSLOD falls below the chosen threshold).
- NON_MENDELIAN – Variant calls are not consistent with Mendelian segregation because one or more progeny have an allele not found in either parent.
- MISSING_PARENT – One or both parents have a missing genotype call.
- NON_SEGREGATING – Variant is fixed within the sample set (not necessarily a spurious variant but a useful filter annotation as most analyses shown here use only segregating variants).
- DUP_SITE – Variant position coincides with another.
- NON_CORE – Variant is not within the core genome.
- LOW_CONFIDENCE_PARENT – Genotype confidence for one or both parents is low (GQ < 99).
- CNV – There is evidence for copy number variation at this locus.

The CNV filter was applied based on evidence from depth of coverage data, described in the section on CNV analysis below.

For all downstream analyses we also treated genotype calls with a genotype quality (GQ) of less than 99 as missing, although this annotation is not included in the VCF files.

Figure S6 illustrates variant calls from the alignment-based method before and after filtering for a single cross and chromosome. Variant calls for all crosses and chromosomes can be browsed via the web application at http://www.malariagen.net/apps/pf-crosses/ both with and without filters. VCF files can be downloaded from the FTP site.

1.3.2. Assembly-based calling method (Cortex)

Cortex calls were made using version 1.0.5.16 (Iqbal et al. 2013) with the independent workflow, using kmer sizes 31 and 61, automatic error cleaning, the bubble-calling algorithm for variant discovery, memory parameters : mem_height=21, mem_width 150, and quality threshold of 1. The population classifier was used to remove hidden paralogs (--apply_pop_classifier flag).

The rate of Mendelian error was plotted against the number of variants for different thresholds of the SITE_CONF score (**Figure S5**). Based on these plots we used a target Mendelian error rate of ~0.05% to decide variant and call filtering strategies. For SNPs we chose a SITE_CONF threshold of 50 and for INDELs we chose a SITE_CONF threshold of 200. These thresholds were the same for all crosses.

We generated a final, analysis-ready VCF for each cross by adding the following filter annotations:

- LOW_CONFIDENCE – Variant confidence is low (SITE_CONF falls below the chosen threshold).
- NON_MENDELIAN – Variant calls are not consistent with Mendelian segregation because one or more progeny have an allele not found in either parent.
- MISSING_PARENT – One or both parents have a missing genotype call.
- NON_SEGREGATING – Variant is fixed within the sample set (not necessarily a spurious variant but a useful filter annotation as most analyses shown here use only segregating variants).
- DUP_SITE – Variant position coincides with another.
- NON_CORE – Variant is not within the core genome.
- LOW_CONFIDENCE_PARENT – Genotype confidence for one or both parents is low (GT_CONF < 50).
- CNV – There is evidence for copy number variation at this locus.

Note that these are in addition to a number of filter annotations previously added as a standard part of the Cortex pipeline.

For all downstream analyses we also treated genotype calls with a GT_CONF of less than 50 as missing, although this annotation is not included in the VCF files.

Figure S7 illustrates variant calls from the assembly-based method before and after filtering for a single cross and chromosome. Variant calls for all crosses and chromosomes can be browsed via the web application at http://www.malariagen.net/apps/pf-crosses/ both with and without filters. VCF files can be downloaded from the FTP site.

1.3.3. Combined callset

A single callset of segregating variants was constructed for each cross by combining variant calls from the alignment and assembly-based methods as follows. For each calling method, a VCF was derived from the full analysis-ready VCF by selecting only variants that passed all filters and segregated within the cross. These two VCFs were then combined into a single VCF using the GATK CombineVariants task, taking genotype calls from the alignment-based calling method where both methods reported the same variant (because the alignment-based method had lower levels of missingness). This produced a single combined VCF of segregating variation for each cross. These VCFs were then post-processed to add a DUP_SITE filter annotation to any variant that coincided with another variant but reported different alleles.

1.3.4. Genotype concordance between biological replicates

In the 3D7xHB3 cross one replicate for clone C01 and 3 replicates for clone C02 were sequenced and genotyped independently. This provided 6 replicate pairs for analysis of genotype concordance. In the 7G8xGB4 cross a single replicate was obtained for each of 10 progeny clones, providing 10 replicate pairs. We computed genotype concordance for each replicate pair and for each of the three available callsets (alignment-based method, assembly-based method, combined) after filtering variants and genotype calls as described above. We computed concordance for each replicate pair as the number of sites where both samples had a matching genotype call divided by the number of sites where both samples had a non-missing genotype call. The results are given in **Table S3**.

1.3.5. Estimation of FDR and sensitivity

To estimate false discovery rate (FDR) and sensitivity, we compared the variant calls generated in this study with pre-existing sequence data resources for the clone HB3. We downloaded contigs from the HB3 draft genome assembly produced from shotgun sequencing by Birren et al. (2006). We also downloaded HB3 sequences for individual genes deposited in GenBank. We aligned both the HB3 contigs and the gene sequences to the 3D7 reference genome using `bwa mem` with the `-x intractg` option (parameters tuned for mapping contigs within a species). We limited further analyses to a set of 32 genes that were completely covered by a single uniquely mapped contig from the draft assembly and by a gene sequence (**Table S4**). In spite of these criteria there remained some considerable discordance between the draft assembly and the gene sequences, particularly regarding INDELs (**Figure S17**). Given that both of these sources may themselves contain errors, we used the following methods to estimate FDR and sensitivity. To estimate FDR we compared variants discovered in this study with the union of variants found in the draft assembly and the gene sequences. Thus a true positive is a variant discovered in this study and also found in either of the other sources, and a false positive is a variant discovered in this study but not present in either of the other sources. To estimate sensitivity we compared variants discovered in this study with the intersection of variants found in the draft assembly and the gene sequences. Thus a false negative is a variant not discovered in this study but present in both of the other sources.

FDR and sensitivity were computed for the replicates HB3(1) and HB3(2) separately, and for each of the two variant calling methods. For INDELs these metrics were computed under two different matching schemes: "position match" where we require the position and type (insertion/deletion) of the variant to match but allow the allele to be different, and "allele match" where we require the position and allele to match perfectly. The results are reported in **Table S5**.

Note that for these comparisons we included all variant alleles called for an HB3 sample, regardless of whether they segregated within a cross (i.e., we ignored the NON_SEGREGATING filter annotations). This is particularly relevant for the HB3(2) sample which was genotyped as part of the HB3xDd2 cross and where many alternate alleles were shared with clone Dd2 and were fixed in all progeny.

We found that the assembly calling method had lower SNP and indel sensitivity for clone HB3(2) compared to HB3(1). This lower sensitivity for HB3(2) was partly due to a technical limitation of the assembly calling method, which was only capable of genotyping variants with a single non-reference allele. Clone HB3(1) was called together with all samples in the 3D7xHB3 cross, and because 3D7 is also the reference clone, only one non-reference haplotype was present. Clone HB3(2) was called with all samples in the HB3xDd2 cross, thus two non-reference haplotypes were present, and some variants would be expected where these haplotypes carry different non-reference alleles. Indeed the alignment method, capable of genotyping any number of alleles, found only 379 INDELs with more than one non-reference allele in the 3D7xHB3 cross, compared with 3732 in HB3xDd2 and 3834 in 7G8xGB4. This limitation does not account for the lower SNP sensitivity, however, as the number of SNPs called by the alignment method with more than one non-reference allele was similar in all three crosses.

The estimated INDEL FDR of 8.3-12.5% seems at odds with the fact that inheritance of SNP and INDEL alleles was highly concordant in all three crosses, and INDEL genotypes were almost perfectly reproducible across multiple biological replicates. If we relaxed the FDR matching condition to require only that variants match type and position, estimated INDEL FDR for the alignment-based method was reduced to 5.3-6.2%. The mismatching alleles were always STR INDELs with the correct type (insertion/deletion) and repeat unit (e.g., "AT") but an incorrect allele length. This could indicate a tendency for the alignment-based method to systematically miscall STR allele length. Some of these mismatches could also be due to genetic variation between HB3 clones with different culturing histories. We also noted considerable discordance between the HB3 draft assembly and published gene sequences regarding INDELs. Although the draft assembly seemed generally more concordant with our variant calls at the 32 genes examined, this was not always the case, and we suspect both the draft assembly and published gene sequences contain INDEL errors. These findings highlight the need for multiple *P. falciparum* genomes fully assembled to the same quality as the current 3D7 reference, so that methods for calling all types of polymorphism and can be accurately evaluated.

1.4. Recombination analyses

1.4.1. Calling CO and NCO recombination events

Two types of recombination event are expected: crossover (CO) and non-crossover (NCO). A CO is a reciprocal exchange accompanied by a conversion tract, whereas a NCO is a conversion tract

without reciprocal exchange (Youds and Boulton 2011). A conversion tract can either be simple (all alleles converted to the same parent) or complex (containing switches between parental alleles converted). In studies of yeast or other organisms where all four daughters of a single meiosis can be captured and genotyped, NCO events can be inferred directly from a non-Mendelian ratio of segregation of alleles. For *P. falciparum* crosses it is not possible to isolate all four daughters of a single meiosis, and thus unequal segregation cannot be directly observed. However, CO and NCO events can be inferred from the patterns of allelic inheritance in the progeny of each cross. Two or more CO events are unlikely to appear in the same progeny clone in close proximity, and thus two or more nearby switches in allelic inheritance are more likely to indicate a conversion tract.

To determine an appropriate threshold for differentiating CO from NCO events, for each cross we first identified contiguous blocks of markers within each progeny where alleles were all inherited from the same parent (parental haplotype blocks). Boundaries between such blocks thus indicate switches in parental inheritance. Each such block has a minimal size, given by the distance between the outer markers within the block, and a maximal size, given by the distance between the markers flanking the block. The distribution of minimal block sizes was plotted for each cross (**Figure S10**). The resulting distributions were bimodal for all three crosses with a minor peak of blocks around ~1kb extending upward to ~10kb. This minor peak would not be expected from CO events, and suggests an expected size range for NCO conversion tracts, although at this stage we have not accounted for complex conversion tracts (which will appear as multiple adjacent short blocks).

To determine a size limit below which to assume that blocks indicate conversion tracts, we computed the number of blocks of a given size that would be expected from CO events alone, using previously published estimates for the CO recombination rate, which should be reasonably accurate given that a high marker resolution is not required to ascertain CO events. Assuming a uniform CO recombination rate of 12 kb/cM (Ranford-Cartwright and Mwangi 2012) we would expect to observe less than 1% of CO events within 10kb of another CO (by the CDF of the exponential distribution). This model is overly simplistic but serves to provide an estimate for the frequency of small block sizes expected from double cross-over events which is conservative because CO interference is likely to reduce further the true probability of observing smaller blocks. We thus assumed that all blocks observed with minimal length shorted than 10kb were either whole or part of conversion tracts.

The following algorithm was then used to call conversion tracts and CO and NCO events from the size and arrangement of parental haplotype blocks. For each progeny clone, genotype calls were used to identify contiguous regions of the genome where all alleles were inherited from the same parent (inheritance blocks) by iterating through variants within a chromosome and recording switches in inheritance between adjacent variants. Any inheritance blocks with minimal length <10kb occurring in isolation were called as simple conversion tracts. Any blocks with minimal length <10kb occurring directly adjacent to each other were merged into a single complex conversion tract. To identify CO events, all genotype calls within conversion tracts were first masked, and remaining switches in parental inheritance were called as CO events. Conversion tracts occurring directly adjacent to a CO were then identified, and the remaining conversion tracts were called as NCO events. Putative conversion tracts supported by a single marker or with a minimal length less than 100bp were excluded from further analyses. This algorithm is similar in motivation

to that used by Samarakoon et al., (2011) but does not depend on a windowed analysis and has greater flexibility for detecting tracts spanning windows.

1.4.2. Estimation of CO and NCO recombination parameters

To estimate genetic map length from the CO recombination rate the identity map functions was used, as marker density was high (~300bp) and thus no adjustment was required to account for the possibility of unobserved double crossovers.

To estimate conversion tract length, we fitted a geometric distribution as suggested by Hilliker et al., (1994). This distribution has a single parameter *phi*, which can be interpreted as the per-base-pair probability of extending a conversion tract, once it has been initiated. To account for the effect of ascertaining conversion tracts via the available SNP and INDEL markers, we simulating 50000 random NCO conversion tracts for each of a range of values for *phi*, then passed the simulated tracts through the same NCO calling process as used for the real data to obtain an empirical distribution for the observed minimal tract length. For each simulation run we compared the resulting distribution of tract lengths with the actual data via a quantile-quantile plot (e.g., Figure 3E) and chose a value of *phi* with the smallest total residual. This process was repeated for each cross separately, and all three crosses gave a close agreement for the value of *phi*.

Once we had fitted the tract length distribution, we then used this information to estimate the NCO discovery rate. This was necessary because we filtered out putative conversion tracts supported by less than two markers spanning less than 100bp, and thus some true conversion tracts will have been filtered, and some conversion tracts may have fallen entirely between markers thus not been observed. We used the NCO simulation described above for *phi* = 0.9993 and for each cross calculated the fraction of simulated NCOs that would have actually been observed using the given markers and the NCO calling process. We then used these estimates for the NCO discovery rate to multiply the observed number of NCO rates per meiosis in each cross by an appropriate factor, to arrive at an estimate for the actual rate of NCO recombination events.

To estimate the CO recombination rate relative to centromere position we divided each chromosome into non-overlapping windows of 20000bp starting from the centromere moving both up and down stream. Within each window we calculated a map length from CO events using the identity map function, then averaging over all windows at each given distance from the centromere we plotted the mean and 95% confidence interval from 1000 bootstrap replicates (Figure 3C).

To estimate the expected number of CO events occurring within genes, intergenic regions, exons and introns we performed Monte Carlo simulations under a null hypothesis of random recombination. In each run a number of CO events for each cross were simulated, then for each CO event the flanking markers were determined according to the markers available in each cross. The number of CO events simulated in each run was equal to the number of CO events observed for each cross. From each run we counted the number of CO events that would have been observed entirely within a gene, entirely within an intergenic region, spanning a gene boundary, etc. We obtained an empirical distribution for these counts from 10000 simulation runs. A similar process was followed for NCO events.

1.4.3. Recombination within CNVs

Read counts were computed for all samples in non-overlapping 300bp windows over the genome, counting the number of reads whose alignment starts within the window, using pysamstats. We observed some GC-related coverage bias in almost all sequencing runs, such that coverage was lower in windows where GC was below 20%. We tried correcting for this bias using the method of Abyzov et al. (2011) however the variance in coverage relative to the median was also higher where GC was below 20% (data not shown) and this correction led to many apparent increases in copy number in low-GC regions, so instead we simply excluded windows where GC content was below 20% from further plots and analyses. To normalise the read count data for each sample we divided the read counts in each window by the median across all windows within the core region of chromosome 14, after visually inspecting the read counts for chromosome 14 in all samples to verify that there was no evidence for copy number variation on that chromosome. These normalised read counts are plotted as black circles in **Figure 3** and **Figure S13**. To view the evidence for the arrangement of amplified segments, depth of coverage was computed within regions of interest for reads aligned facing away from their mate pair ("face-away", indicating a tandem array) and for reads aligned in the same orientation as their mate pair (indicating a tandem inversion), using `pysamstats`. These data are plotted as purple and green lines in **Figure 3** and **Figure S13**.

We also fitted a model of copy number state to the normalised read count data, to aid in visualising the copy number changes in plots. For each sample we fitted a Gaussian HMM using the `scikit-learn` package[2]. Parameters for the model were tuned to minimise prediction of copy number changes within the core genome for clone 3D7 (also the reference clone) and to minimise prediction of copy number changes that do not segregate in both parents and progeny of a cross, assuming that these two conditions are both indicative of false positive predictions of copy number change, and to maximise sensitivity for detecting known copy number amplifications at the *mdr1* and *gch1* loci. A complete worked example is given at http://nbviewer.ipython.org/github/alimanfoo/hmmcnv/blob/master/tutorial.ipynb.

We also used the fitted HMM model to scan the core genome for other loci with evidence of copy number change. Apart from the *mdr1* and *gch1* loci, the only other loci with evidence for copy number change spanning one or more genes were a deletion at the *clag3* locus (Chung et al. 2007; Sepúlveda et al. 2013) present in GB4 and segregating in the 7G8xGB4 cross, and a translocated duplication adjacent to the subtelomere of chromosome 11 (Hinterberg et al. 1994) present in both HB3 replicates and segregating in the HB3xDd2 progeny.

To examine evidence for regions of pseudo-heterozygosity at the *gch1* locus in progeny clones C05, C06 and CH3_61 we used the raw allele depths (AD) emitted by GATK at variant sites and plotted the ratio of allele depths for the first parent's allele (e.g., 3D7 for clones C05 and C06) divided by the total depth of coverage.

1.5. A Web application to facilitate data exploration and re-use

The sequence and variation data generated in this study are a rich resource and could serve many purposes beyond the analyses presented here. To facilitate re-use of these data we developed a Web application that provides a number of novel tools for intuitive, interactive data exploration,

2 http://scikit-learn.org

available at www.malariagen.net/apps/pf-crosses. The introduction page (Figure S19A) provides navigation to a set of tools, including a tool for browsing and querying a table of variants for each cross and calling method (Figure S19B); a tool for visualising and browsing the genotype calls at individual samples and patterns of inheritance and recombination within each cross (Figure S19C); a tool for browsing the genome, allowing the location of variants to be viewed in the context of genome features and alignment metrics (Figure S19D); and a browser for visualising the sequence alignments themselves, implemented by embedding the LookSeq software (Manske and Kwiatkowski 2009) (Fig S19E). All tools are highly interactive, for example when browsing genotypes the user can hover over any variant and view further information about the reference and alternate alleles, effect prediction, etc. Filters applied to variants can also be changed dynamically, allowing users to explore the entire dataset and compare calling and filtering methods. For the genome browser, a multi-resolution filterbank was implemented to enable highly responsive browsing at all scales from base-pair resolution up to entire chromosomes. The underlying technologies for this Web application are being developed as a generic framework so that they can be used with other organisms and datasets, as part of the open source Panoptes project[3].

3 https://github.com/cggh/panoptes

2. Supplemental tables

Index of tables

Table S1..14
Table S2..16
Table S3..18
Table S4..19
Table S5..20

***Table S1**: Samples and sequencing runs generated in this study.*

Cross	Clone	Sample	Run	Instrument	Coverage
3D7 x HB3	3D7	PG0051-C	ERR019061	Illumina Genome Analyzer II	122X
3D7 x HB3	C01	PG0065-C	ERR019064	Illumina Genome Analyzer II	163X
3D7 x HB3	C01	PG0062-C	ERR019070	Illumina Genome Analyzer II	108X
3D7 x HB3	C02	PG0055-C	ERR019066	Illumina Genome Analyzer II	102X
3D7 x HB3	C02	PG0053-C	ERR019067	Illumina Genome Analyzer II	73X
3D7 x HB3	C02	PG0056-C	ERR019068	Illumina Genome Analyzer II	84X
3D7 x HB3	C02	PG0067-C	ERR019073	Illumina Genome Analyzer II	126X
3D7 x HB3	C03	PG0066-C	ERR019072	Illumina Genome Analyzer II	79X
3D7 x HB3	C04	PG0061-C	ERR019059	Illumina Genome Analyzer II	165X
3D7 x HB3	C05	PG0068-C	ERR019065	Illumina Genome Analyzer II	41X
3D7 x HB3	C06	PG0069-C	ERR019055	Illumina Genome Analyzer II	135X
3D7 x HB3	C07	PG0070-C	ERR019056	Illumina Genome Analyzer II	144X
3D7 x HB3	C08	PG0071-C	ERR019074	Illumina Genome Analyzer II	120X
3D7 x HB3	C09	PG0072-C	ERR019057	Illumina Genome Analyzer II	173X
3D7 x HB3	C10	PG0063-C	ERR019060	Illumina Genome Analyzer II	108X
3D7 x HB3	C11	PG0064-C	ERR019071	Illumina Genome Analyzer II	48X
3D7 x HB3	C12	PG0058-C	ERR019063	Illumina Genome Analyzer II	51X
3D7 x HB3	C13	PG0054-C	ERR019062	Illumina Genome Analyzer II	95X
3D7 x HB3	C14	PG0060-C	ERR019058	Illumina Genome Analyzer II	102X
3D7 x HB3	C15	PG0057-C	ERR019069	Illumina Genome Analyzer II	56X
3D7 x HB3	HB3	PG0052-C	ERR019054	Illumina Genome Analyzer II	100X
7G8 x GB4	7G8	PG0083-C	ERR027099	Illumina Genome Analyzer II	87X
7G8 x GB4	AL2	PG0103-CW	ERR045627	Illumina HiSeq 2000	127X
7G8 x GB4	AUD	PG0112-C	ERR029406	Illumina Genome Analyzer II	129X
7G8 x GB4	AUD	PG0112-CW	ERR045639	Illumina HiSeq 2000	88X
7G8 x GB4	D2	PG0094-CW	ERR045632	Illumina HiSeq 2000	153X
7G8 x GB4	DAN	PG0098-C	ERR027110	Illumina Genome Analyzer II	140X
7G8 x GB4	DEV	PG0081-CW	ERR045633	Illumina HiSeq 2000	89X
7G8 x GB4	GB4	PG0084-C	ERR027100	Illumina Genome Analyzer II	104X
7G8 x GB4	JB12	PG0099-C	ERR029146	Illumina Genome Analyzer II	120X
7G8 x GB4	JB8	PG0087-C	ERR029091	Illumina Genome Analyzer II	103X
7G8 x GB4	JC3	PG0077-CW	ERR045636	Illumina HiSeq 2000	94X
7G8 x GB4	JC9	PG0111-C	ERR029409	Illumina Genome Analyzer II	122X
7G8 x GB4	JC9	PG0111-CW	ERR045634	Illumina HiSeq 2000	121X
7G8 x GB4	JE11	PG0100-C	ERR029404	Illumina Genome Analyzer II	134X
7G8 x GB4	JE11	PG0100-CW	ERR045630	Illumina HiSeq 2000	55X
7G8 x GB4	JF6	PG0079-C	ERR027102	Illumina Genome Analyzer II	181X
7G8 x GB4	JF6	PG0079-CW	ERR045637	Illumina HiSeq 2000	94X
7G8 x GB4	JON	PG0107-C	ERR029408	Illumina Genome Analyzer II	180X
7G8 x GB4	KA6	PG0091-C	ERR027117	Illumina Genome Analyzer II	80X
7G8 x GB4	KB8	PG0104-C	ERR029148	Illumina Genome Analyzer II	116X
7G8 x GB4	KB8	PG0104-CW	ERR045642	Illumina HiSeq 2000	81X
7G8 x GB4	KH7	PG0088-C	ERR027111	Illumina Genome Analyzer II	96X
7G8 x GB4	LA10	PG0086-C	ERR029090	Illumina Genome Analyzer II	119X
7G8 x GB4	LA10	PG0086-CW	ERR045629	Illumina HiSeq 2000	66X
7G8 x GB4	NF10	PG0096-C	ERR027108	Illumina Genome Analyzer II	75X
7G8 x GB4	NIC	PG0095-C	ERR027107	Illumina Genome Analyzer II	70X
7G8 x GB4	NIC	PG0095-CW	ERR045631	Illumina HiSeq 2000	80X
7G8 x GB4	QF5	PG0078-C	ERR029092	Illumina Genome Analyzer II	147X
7G8 x GB4	QF5	PG0078-CW	ERR045638	Illumina HiSeq 2000	82X
7G8 x GB4	TF1	PG0080-C	ERR027103	Illumina Genome Analyzer II	73X
7G8 x GB4	WC4	PG0082-C	ERR029093	Illumina Genome Analyzer II	78X
7G8 x GB4	WE2	PG0085-C	ERR027101	Illumina Genome Analyzer II	124X
7G8 x GB4	WF12	PG0097-C	ERR027109	Illumina Genome Analyzer II	109X
7G8 x GB4	XB3	PG0093-C	ERR029105	Illumina Genome Analyzer II	214X
7G8 x GB4	XD8	PG0105-C	ERR029144	Illumina Genome Analyzer II	121X
7G8 x GB4	XD8	PG0105-CW	ERR045628	Illumina HiSeq 2000	122X

Cross	Clone	Sample	Run	Instrument	Coverage
7G8 x GB4	XE7	PG0106-C	ERR029407	Illumina Genome Analyzer II	250X
7G8 x GB4	XF12	PG0102-C	ERR029143	Illumina Genome Analyzer II	141X
7G8 x GB4	XF12	PG0102-CW	ERR045635	Illumina HiSeq 2000	96X
7G8 x GB4	XG10	PG0109-C	ERR029405	Illumina Genome Analyzer II	61X
HB3 x Dd2	1BB5	PG0023-C	ERR015449	Illumina Genome Analyzer II	22X
HB3 x Dd2	3BA6	PG0022-Cx	ERR126027	Illumina HiSeq 2000	32X
HB3 x Dd2	3BD5	PG0024-C	ERR019053	Illumina Genome Analyzer II	92X
HB3 x Dd2	7C101	PG0074-C	ERR019048	Illumina Genome Analyzer II	98X
HB3 x Dd2	7C111	PG0038-C	ERR015457	Illumina Genome Analyzer II	148X
HB3 x Dd2	7C12	PG0035-Cx	ERR037704	Illumina HiSeq 2000	637X
HB3 x Dd2	7C126	PG0047-C	ERR015452	Illumina Genome Analyzer II	187X
HB3 x Dd2	7C140	PG0039-C	ERR015454	Illumina Genome Analyzer II	78X
HB3 x Dd2	7C159	PG0040-Cx	ERR107475	Illumina HiSeq 2000	59X
HB3 x Dd2	7C16	PG0036-C	ERR015455	Illumina Genome Analyzer II	26X
HB3 x Dd2	7C170	PG0041-C	ERR015446	Illumina Genome Analyzer II	130X
HB3 x Dd2	7C183	PG0042-C	ERR015448	Illumina Genome Analyzer II	118X
HB3 x Dd2	7C188	PG0030-C	ERR019046	Illumina Genome Analyzer II	171X
HB3 x Dd2	7C20	PG0037-C	ERR015451	Illumina Genome Analyzer II	82X
HB3 x Dd2	7C3	PG0034-C	ERR019047	Illumina Genome Analyzer II	142X
HB3 x Dd2	7C408	PG0031-C	ERR015458	Illumina Genome Analyzer II	51X
HB3 x Dd2	7C421	PG0043-C	ERR015459	Illumina Genome Analyzer II	164X
HB3 x Dd2	7C424	PG0044-C	ERR019043	Illumina Genome Analyzer II	172X
HB3 x Dd2	7C46	PG0046-Cx	ERR107476	Illumina HiSeq 2000	62X
HB3 x Dd2	7C7	PG0048-C	ERR019049	Illumina Genome Analyzer II	110X
HB3 x Dd2	B1SD	PG0015-C	ERR019044	Illumina Genome Analyzer II	91X
HB3 x Dd2	B4R3	PG0018-C	ERR019042	Illumina Genome Analyzer II	115X
HB3 x Dd2	CH3_116	PG0032-Cx	ERR037703	Illumina HiSeq 2000	186X
HB3 x Dd2	CH3_61	PG0033-Cx	ERR175544	Illumina HiSeq 2000	68X
HB3 x Dd2	D43	PG0029-Cx	ERR107474	Illumina HiSeq 2000	34X
HB3 x Dd2	DD2	PG0008-CW	ERR012840	Illumina Genome Analyzer II	122X
HB3 x Dd2	GC03	PG0021-C	ERR015447	Illumina Genome Analyzer II	152X
HB3 x Dd2	GC06	PG0028-C	ERR015456	Illumina Genome Analyzer II	54X
HB3 x Dd2	HB3	PG0004-CW	ERR012788	Illumina Genome Analyzer II	80X
HB3 x Dd2	QC01	PG0017-C	ERR019050	Illumina Genome Analyzer II	117X
HB3 x Dd2	QC13	PG0016-C	ERR012895	Illumina Genome Analyzer II	68X
HB3 x Dd2	QC23	PG0045-C	ERR012892	Illumina Genome Analyzer II	115X
HB3 x Dd2	QC34	PG0026-C	ERR015453	Illumina Genome Analyzer II	55X
HB3 x Dd2	SC01	PG0025-C	ERR019045	Illumina Genome Analyzer II	149X
HB3 x Dd2	SC05	PG0019-C	ERR019051	Illumina Genome Analyzer II	97X
HB3 x Dd2	TC05	PG0027-C	ERR015450	Illumina Genome Analyzer II	115X
HB3 x Dd2	TC08	PG0020-C	ERR019052	Illumina Genome Analyzer II	144X

***Table S2**: Genome region classification.*

Chromosome	Start	Stop	Type	Size
Pf3D7_01_v3	1	27336	SubtelomericRepeat	27336
Pf3D7_01_v3	27337	92900	SubtelomericHypervariable	65564
Pf3D7_01_v3	92901	457931	Core	365031
Pf3D7_01_v3	457932	460311	Centromere	2380
Pf3D7_01_v3	460312	575900	Core	115589
Pf3D7_01_v3	575901	616691	SubtelomericHypervariable	40791
Pf3D7_01_v3	616692	640851	SubtelomericRepeat	24160
Pf3D7_02_v3	1	23100	SubtelomericRepeat	23100
Pf3D7_02_v3	23101	105800	SubtelomericHypervariable	82700
Pf3D7_02_v3	105801	447300	Core	341500
Pf3D7_02_v3	447301	450450	Centromere	3150
Pf3D7_02_v3	450451	862500	Core	412050
Pf3D7_02_v3	862501	925850	SubtelomericHypervariable	63350
Pf3D7_02_v3	925851	947102	SubtelomericRepeat	21252
Pf3D7_03_v3	1	34268	SubtelomericRepeat	34268
Pf3D7_03_v3	34269	70630	SubtelomericHypervariable	36362
Pf3D7_03_v3	70631	597816	Core	527186
Pf3D7_03_v3	597817	600275	Centromere	2459
Pf3D7_03_v3	600276	1003060	Core	402785
Pf3D7_03_v3	1003061	1040961	SubtelomericHypervariable	37901
Pf3D7_03_v3	1040962	1067971	SubtelomericRepeat	27010
Pf3D7_04_v3	1	26511	SubtelomericRepeat	26511
Pf3D7_04_v3	26512	91420	SubtelomericHypervariable	64909
Pf3D7_04_v3	91421	545800	Core	454380
Pf3D7_04_v3	545801	614900	InternalHypervariable	69100
Pf3D7_04_v3	614901	642003	Core	27103
Pf3D7_04_v3	642004	644529	Centromere	2526
Pf3D7_04_v3	644530	935030	Core	290501
Pf3D7_04_v3	935031	983080	InternalHypervariable	48050
Pf3D7_04_v3	983081	1143990	Core	160910
Pf3D7_04_v3	1143991	1182297	SubtelomericHypervariable	38307
Pf3D7_04_v3	1182298	1200490	SubtelomericRepeat	18193
Pf3D7_05_v3	1	18750	SubtelomericRepeat	18750
Pf3D7_05_v3	18751	37900	SubtelomericHypervariable	19150
Pf3D7_05_v3	37901	455740	Core	417840
Pf3D7_05_v3	455741	457252	Centromere	1512
Pf3D7_05_v3	457253	1321390	Core	864138
Pf3D7_05_v3	1321391	1342974	SubtelomericHypervariable	21584
Pf3D7_05_v3	1342975	1343577	SubtelomericRepeat	603
Pf3D7_06_v3	1	610	SubtelomericRepeat	610
Pf3D7_06_v3	611	72350	SubtelomericHypervariable	71740
Pf3D7_06_v3	72351	478652	Core	406302
Pf3D7_06_v3	478653	480971	Centromere	2319
Pf3D7_06_v3	480972	723117	Core	242146
Pf3D7_06_v3	723118	742800	InternalHypervariable	19683
Pf3D7_06_v3	742801	1294830	Core	552030
Pf3D7_06_v3	1294831	1384651	SubtelomericHypervariable	89821
Pf3D7_06_v3	1384652	1418242	SubtelomericRepeat	33591
Pf3D7_07_v3	1	18000	SubtelomericRepeat	18000
Pf3D7_07_v3	18001	77100	SubtelomericHypervariable	59100
Pf3D7_07_v3	77101	508360	Core	431260
Pf3D7_07_v3	508361	605650	InternalHypervariable	97290
Pf3D7_07_v3	605651	809245	Core	203595
Pf3D7_07_v3	809246	811716	Centromere	2471
Pf3D7_07_v3	811717	1381600	Core	569884
Pf3D7_07_v3	1381601	1428410	SubtelomericHypervariable	46810
Pf3D7_07_v3	1428411	1445207	SubtelomericRepeat	16797

Chromosome	Start	Stop	Type	Size
Pf3D7_08_v3	1	19100	SubtelomericRepeat	19100
Pf3D7_08_v3	19101	73560	SubtelomericHypervariable	54460
Pf3D7_08_v3	73561	299079	Core	225519
Pf3D7_08_v3	299080	301403	Centromere	2324
Pf3D7_08_v3	301404	427430	Core	126027
Pf3D7_08_v3	427431	467340	InternalHypervariable	39910
Pf3D7_08_v3	467341	1365730	Core	898390
Pf3D7_08_v3	1365731	1445690	SubtelomericHypervariable	79960
Pf3D7_08_v3	1445691	1472805	SubtelomericRepeat	27115
Pf3D7_09_v3	1	17955	SubtelomericRepeat	17955
Pf3D7_09_v3	17956	79100	SubtelomericHypervariable	61145
Pf3D7_09_v3	79101	1242137	Core	1163037
Pf3D7_09_v3	1242138	1244483	Centromere	2346
Pf3D7_09_v3	1244484	1473560	Core	229077
Pf3D7_09_v3	1473561	1505792	SubtelomericHypervariable	32232
Pf3D7_09_v3	1505793	1541735	SubtelomericRepeat	35943
Pf3D7_10_v3	1	26240	SubtelomericRepeat	26240
Pf3D7_10_v3	26241	68970	SubtelomericHypervariable	42730
Pf3D7_10_v3	68971	1571815	Core	1502845
Pf3D7_10_v3	1571816	1652190	SubtelomericHypervariable	80375
Pf3D7_10_v3	1652191	1687656	SubtelomericRepeat	35466
Pf3D7_11_v3	1	21990	SubtelomericRepeat	21990
Pf3D7_11_v3	21991	110000	SubtelomericHypervariable	88010
Pf3D7_11_v3	110001	831968	Core	721968
Pf3D7_11_v3	831969	834245	Centromere	2277
Pf3D7_11_v3	834246	2003320	Core	1169075
Pf3D7_11_v3	2003321	2037033	SubtelomericHypervariable	33713
Pf3D7_11_v3	2037034	2038340	SubtelomericRepeat	1307
Pf3D7_12_v3	1	14780	SubtelomericRepeat	14780
Pf3D7_12_v3	14781	60300	SubtelomericHypervariable	45520
Pf3D7_12_v3	60301	766654	Core	706354
Pf3D7_12_v3	766655	780450	InternalHypervariable	13796
Pf3D7_12_v3	780451	1282773	Core	502323
Pf3D7_12_v3	1282774	1285067	Centromere	2294
Pf3D7_12_v3	1285068	1688600	Core	403533
Pf3D7_12_v3	1688601	1745530	InternalHypervariable	56930
Pf3D7_12_v3	1745531	2163700	Core	418170
Pf3D7_12_v3	2163701	2251150	SubtelomericHypervariable	87450
Pf3D7_12_v3	2251151	2271494	SubtelomericRepeat	20344
Pf3D7_13_v3	1	19160	SubtelomericRepeat	19160
Pf3D7_13_v3	19161	74413	SubtelomericHypervariable	55253
Pf3D7_13_v3	74414	1168127	Core	1093714
Pf3D7_13_v3	1168128	1170425	Centromere	2298
Pf3D7_13_v3	1170426	2791900	Core	1621475
Pf3D7_13_v3	2791901	2894620	SubtelomericHypervariable	102720
Pf3D7_13_v3	2894621	2925236	SubtelomericRepeat	30616
Pf3D7_14_v3	1	1344	SubtelomericRepeat	1344
Pf3D7_14_v3	1345	35774	SubtelomericHypervariable	34430
Pf3D7_14_v3	35775	1071523	Core	1035749
Pf3D7_14_v3	1071524	1075089	Centromere	3566
Pf3D7_14_v3	1075090	3255710	Core	2180621
Pf3D7_14_v3	3255711	3291511	SubtelomericHypervariable	35801
Pf3D7_14_v3	3291512	3291936	SubtelomericRepeat	425

***Table S3**: Genotype discordance between biological replicates. Each row reports discordance data for a single replicate pair. Values given for each callset are [number of variants with a discordant genotype call]/[total number of variants with non-missing genotype calls in both members of the pair].*

			Genotype discordance		
**Cross**	**Clone**	**Replicate pair**	**BWA/GATK callset**	**Cortex callset**	**Combined callset**
3D7 x HB3	C01	C01/PG0062-C/ERR019070 vs C01/PG0065-C/ERR019064	3/36567	1/27152	3/42021
3D7 x HB3	C02	C02/PG0053-C/ERR019067 vs C02/PG0055-C/ERR019066	1/36551	0/27008	1/41977
3D7 x HB3	C02	C02/PG0053-C/ERR019067 vs C02/PG0056-C/ERR019068	1/36530	0/26943	1/41948
3D7 x HB3	C02	C02/PG0053-C/ERR019067 vs C02/PG0067-C/ERR019073	3/36569	0/27068	3/42010
3D7 x HB3	C02	C02/PG0055-C/ERR019066 vs C02/PG0056-C/ERR019068	2/36527	0/27022	2/41949
3D7 x HB3	C02	C02/PG0055-C/ERR019066 vs C02/PG0067-C/ERR019073	5/36573	1/27172	6/42029
3D7 x HB3	C02	C02/PG0056-C/ERR019068 vs C02/PG0067-C/ERR019073	1/36545	0/27090	1/41985
7G8 x GB4	AUD	AUD/PG0112-C/ERR029406 vs AUD/PG0112-CW/ERR045639	32/27524	7/22423	15/33814
7G8 x GB4	JC9	JC9/PG0111-C/ERR029409 vs JC9/PG0111-CW/ERR045634	28/27556	8/22700	8/33998
7G8 x GB4	JE11	JE11/PG0100-C/ERR029404 vs JE11/PG0100-CW/ERR045630	30/27182	2/20800	9/32703
7G8 x GB4	JF6	JF6/PG0079-C/ERR027102 vs JF6/PG0079-CW/ERR045637	25/27529	8/22544	10/33878
7G8 x GB4	KB8	KB8/PG0104-C/ERR029148 vs KB8/PG0104-CW/ERR045642	25/27256	6/21939	13/33296
7G8 x GB4	LA10	LA10/PG0086-C/ERR029090 vs LA10/PG0086-CW/ERR045629	26/27393	2/21724	11/33365
7G8 x GB4	NIC	NIC/PG0095-C/ERR027107 vs NIC/PG0095-CW/ERR045631	32/26991	3/19531	10/31909
7G8 x GB4	QF5	QF5/PG0078-C/ERR029092 vs QF5/PG0078-CW/ERR045638	34/27422	6/22349	18/33682
7G8 x GB4	XD8	XD8/PG0105-C/ERR029144 vs XD8/PG0105-CW/ERR045628	29/27562	13/22572	17/33917
7G8 x GB4	XF12	XF12/PG0102-C/ERR029143 vs XF12/PG0102-CW/ERR045635	32/27507	5/22459	18/33801

Table S4*: Genes used for the estimation of FDR and sensitivity.*

Chromosome	Start	Stop	ID	Name	Previous ID	Genbank Accession
Pf3D7_01_v3	265208	269173	PF3D7_0106300	ATP6	PFA0310c	gi|56342158|dbj|AB121052.1|
Pf3D7_02_v3	290168	292703	PF3D7_0207300	SERA8	PFB0325c	gi|803375251|dbj|AB733715.1|
Pf3D7_02_v3	294273	297616	PF3D7_0207400	SERA7	PFB0330c	gi|803375249|dbj|AB733714.1|
Pf3D7_02_v3	298897	302564	PF3D7_0207500	SERA6	PFB0335c	gi|803375247|dbj|AB733713.1|
Pf3D7_02_v3	303593	307027	PF3D7_0207600	SERA5	PFB0340c	gi|803375245|dbj|AB733712.1|
Pf3D7_02_v3	308847	312155	PF3D7_0207700	SERA4	PFB0345c	gi|803375243|dbj|AB733711.1|
Pf3D7_02_v3	313449	316741	PF3D7_0207800	SERA3	PFB0350c	gi|803375241|dbj|AB733710.1|
Pf3D7_02_v3	322338	325723	PF3D7_0208000	SERA1	PFB0360c	gi|803375237|dbj|AB733708.1|
Pf3D7_03_v3	221323	222516	PF3D7_0304600	CSP	PFC0210c	gi|56342142|dbj|AB121018.1|
Pf3D7_04_v3	137640	146653	PF3D7_0402300	RH1	PFD0110w	gi|33414602|gb|AF411930.2|
Pf3D7_04_v3	748088	749914	PF3D7_0417200	DHFR-TS	PFD0830w	gi|340507|gb|J03772.1|PFADHFRTSE
Pf3D7_04_v3	1085979	1091277	PF3D7_0424200	RH4	PFD1150c	gi|21321386|gb|AF420310.1|
Pf3D7_05_v3	328666	329715	PF3D7_0508000	P38	PFE0395c	gi|133900606|gb|EF137222.1|
Pf3D7_06_v3	851378	852955	PF3D7_0620400	MSP10	PFF0995c	gi|237664869|gb|FJ406615.1|
Pf3D7_07_v3	381592	384614	PF3D7_0708400	HSP90	PF07_0029	gi|505339|gb|L34028.1|PFAHSP86B
Pf3D7_07_v3	408215	411961	PF3D7_0709100		PF07_0035	gi|2642510|gb|AF030690.1|
Pf3D7_07_v3	413560	421749	PF3D7_0709300		PF07_0037	gi|2642515|gb|AF030693.1|
Pf3D7_08_v3	278381	279034	PF3D7_0804800	CYP24	PF08_0121	gi|1000520|gb|U10322.1|PFU10322
Pf3D7_08_v3	1358314	1363618	PF3D7_0831600	CLAG8	MAL7P1.229	gi|167962700|dbj|AB250802.1|
Pf3D7_09_v3	121621	125006	PF3D7_0902800	SERA9	PFI0135c	gi|803375253|dbj|AB733716.1|
Pf3D7_09_v3	270740	274789	PF3D7_0905400	RhopH3	PFI0265c	gi|167962547|dbj|AB250806.1|
Pf3D7_09_v3	1175203	1180762	PF3D7_0929400	RhopH2	PFI1445w	gi|167963178|dbj|AB250805.1|
Pf3D7_09_v3	1413840	1419754	PF3D7_0935800	CLAG9	PFI1730w	gi|167962308|dbj|AB250804.1|
Pf3D7_11_v3	592130	593584	PF3D7_1115700		PF11_0165	gi|9719453|gb|AF282979.1|
Pf3D7_11_v3	1293856	1295724	PF3D7_1133400	AMA1	PF11_0344	gi|182407599|gb|EU586393.1|
Pf3D7_12_v3	1915749	1917798	PF3D7_1246100	ALAS	PFL2210w	gi|1220442|gb|L46348.1|PFADAAS
Pf3D7_13_v3	975403	977175	PF3D7_1323500	PMV	PF13_0133	gi|58372444|gb|AY878742.1|
Pf3D7_13_v3	1416316	1417458	PF3D7_1335000	MSRP1	PF13_0196	gi|237665051|gb|FJ406706.1|
Pf3D7_13_v3	1419086	1420141	PF3D7_1335100	MSP7	PF13_0197	gi|116109338|gb|DQ987539.1|
Pf3D7_13_v3	1497877	1501494	PF3D7_1337200		MAL13P1.186	gi|6690111|gb|AF111814.2|
Pf3D7_14_v3	1368815	1369796	PF3D7_1434200	CAM	PF14_0323	gi|160125|gb|M59349.1|PFACALMOD
Pf3D7_14_v3	1954601	1957675	PF3D7_1447900	MDR2	PF14_0455	gi|294166|gb|L13381.1|PFAMDR2X

***Table S5**: FDR and sensitivity estimates for the two replicate samples of clone HB3. See supplementary text for estimation methods. TP = true positive; FP = false positive; FN = false negative; FDR = false discovery rate.*

Sample	Callset	Variant Type	TP	FP	FN	FDR	Sensitivity
HB3(1)	BWA/GATK	SNPs	178	5	33	2.7%	84.4%
		INDELs (position match)	45	3	18	6.2%	71.4%
		INDELs (allele match)	42	6	18	12.5%	70.0%
	Cortex	SNPs	188	2	22	1.1%	89.5%
		INDELs (position match)	38	4	15	9.5%	71.7%
		INDELs (allele match)	38	4	12	9.5%	76.0%
HB3(2)	BWA/GATK	SNPs	171	1	39	0.6%	81.4%
		INDELs (position match)	36	2	21	5.3%	63.2%
		INDELs (allele match)	34	4	19	10.5%	64.2%
	Cortex	SNPs	57	0	137	0.0%	29.4%
		INDELs (position match)	11	1	35	8.3%	23.9%
		INDELs (allele match)	11	1	29	8.3%	27.5%

3. Supplemental figures

Index of figures

Figure S1....21
Figure S2....22
Figure S3....23
Figure S4....24
Figure S5....25
Figure S6....26
Figure S7....27
Figure S8....28
Figure S9....29
Figure S10....30
Figure S11....31
Figure S12....32
Figure S13....33
Figure S14....34
Figure S15....35
Figure S16....36
Figure S17....37
Figure S18....38
Figure S19....39

***Figure S1**: Example of alignment metrics for an individual sample and relationship to genome region classification. The sample shown is HB3/PG0052-C/ERR019054 (parent of 3D7xHB3) and data are shown for the entirety of chromosome 4. DP = total depth of coverage, PP = percent of reads aligned in a proper pair; MQ = root mean square mapping quality of aligned reads; MQ0 = percent of reads aligned ambiguously (mapping quality zero); MIS = percent of reads aligned with a base mismatching the reference. Genes tracks shows forward strand above the line, reverse strand below the line; genes in red are var/rif/stevor. Genome region classification is shown in the bottom track, colours as in the legend.*

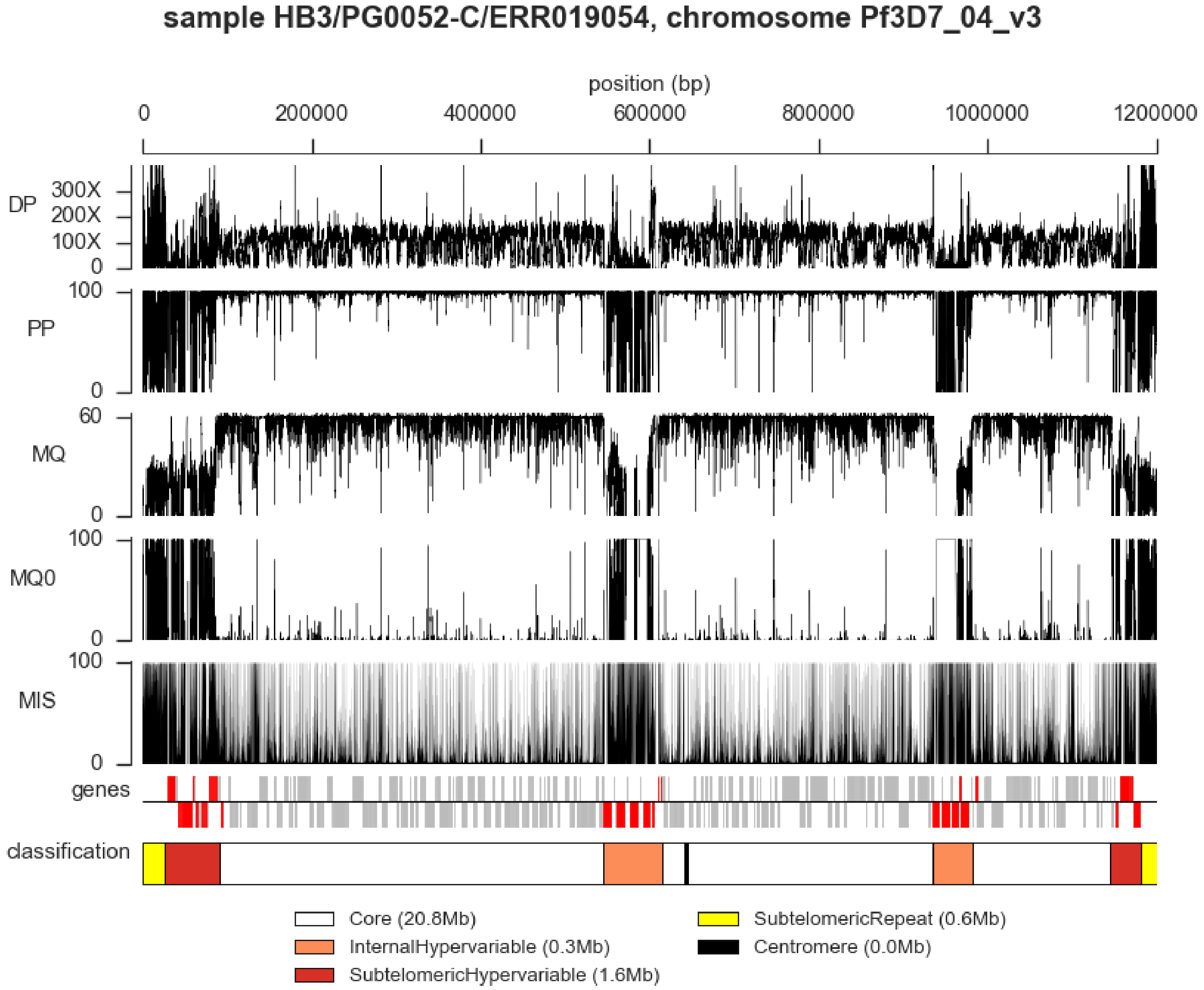

***Figure S2**: Genome region classification. Each sub-plot corresponds to one of the fourteen nuclear chromosomes. The central bar in each sub-plot shows the genome region classification coloured according to the legend. Above the central bar in purple are levels of heterochromatin protein 1 (HP1) per gene from (Flueck et al., 2009). Below in grey are genes, with positive and negative strands plotted above and below the line respectively; genes in the rif, stevor and var families are shown in red.*

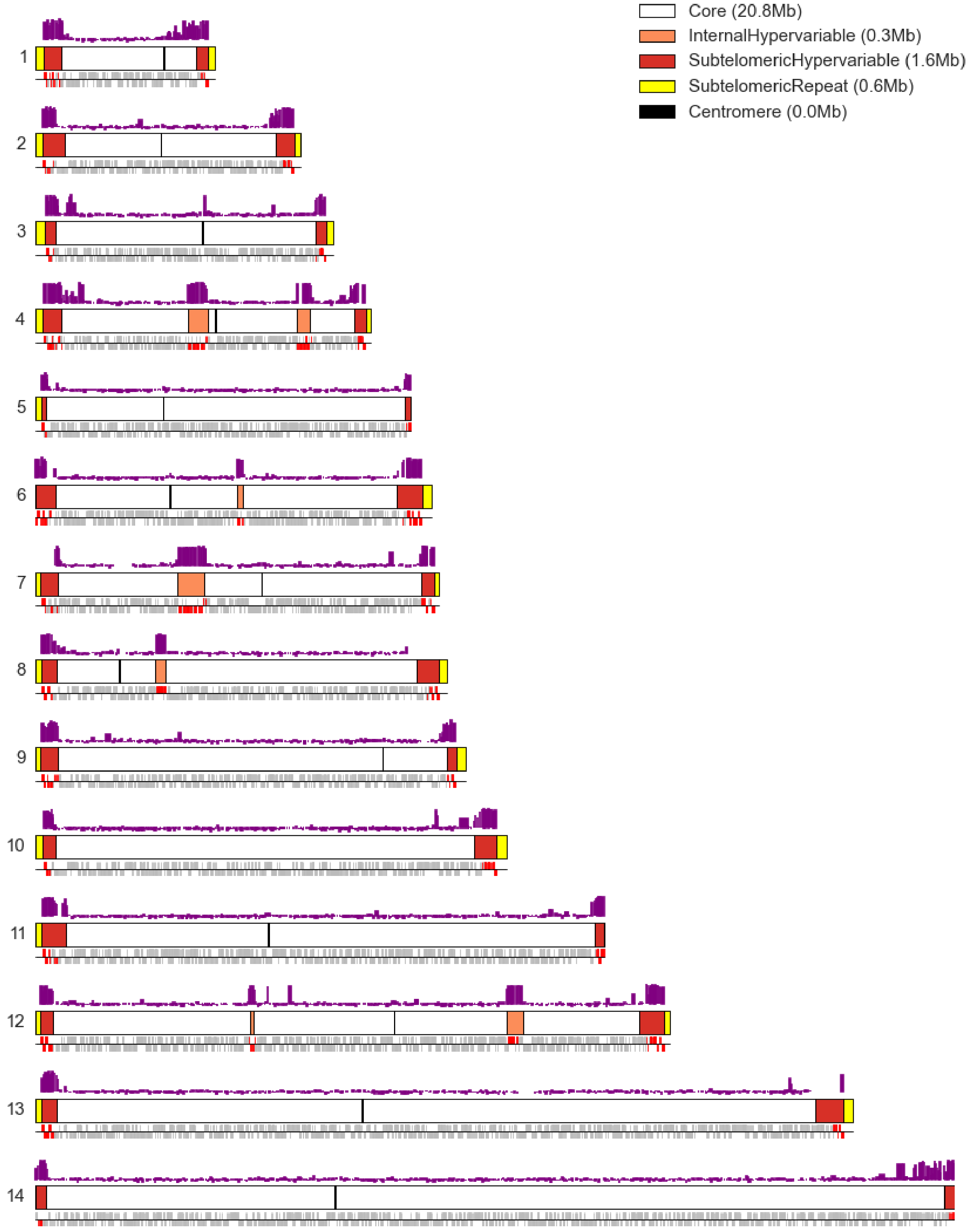

Figure S3*: Summary of alignment characteristics for different genome region classes. The left-hand sub-plot shows the percentage of positions with more than 10% of reads aligned ambiguously (mapping quality zero). The right-hand sub-plot shows the percentage of positions without any coverage whatsoever.*

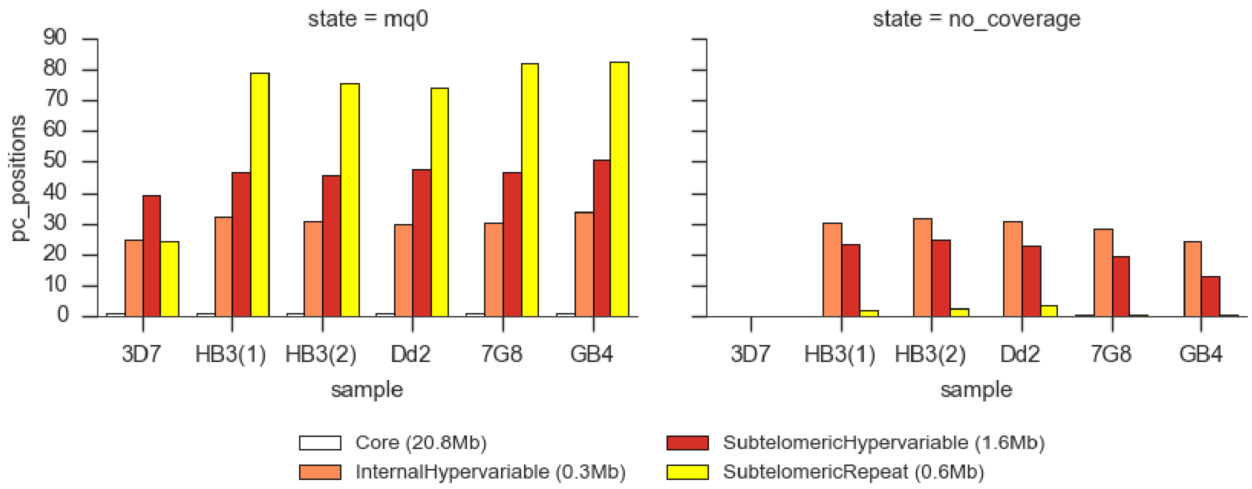

Figure S4: *Using Mendelian error as a guide to filtering variants and genotype calls from the alignment-based calling method. Each point plotted corresponds to variants filtered according to a minimum value of the VQSLOD annotation and genotype calls filtered according to a minimum value of GQ. The VQSLOD threshold value is shown labelling the point, the colour indicates the GQ threshold according to the legend.*

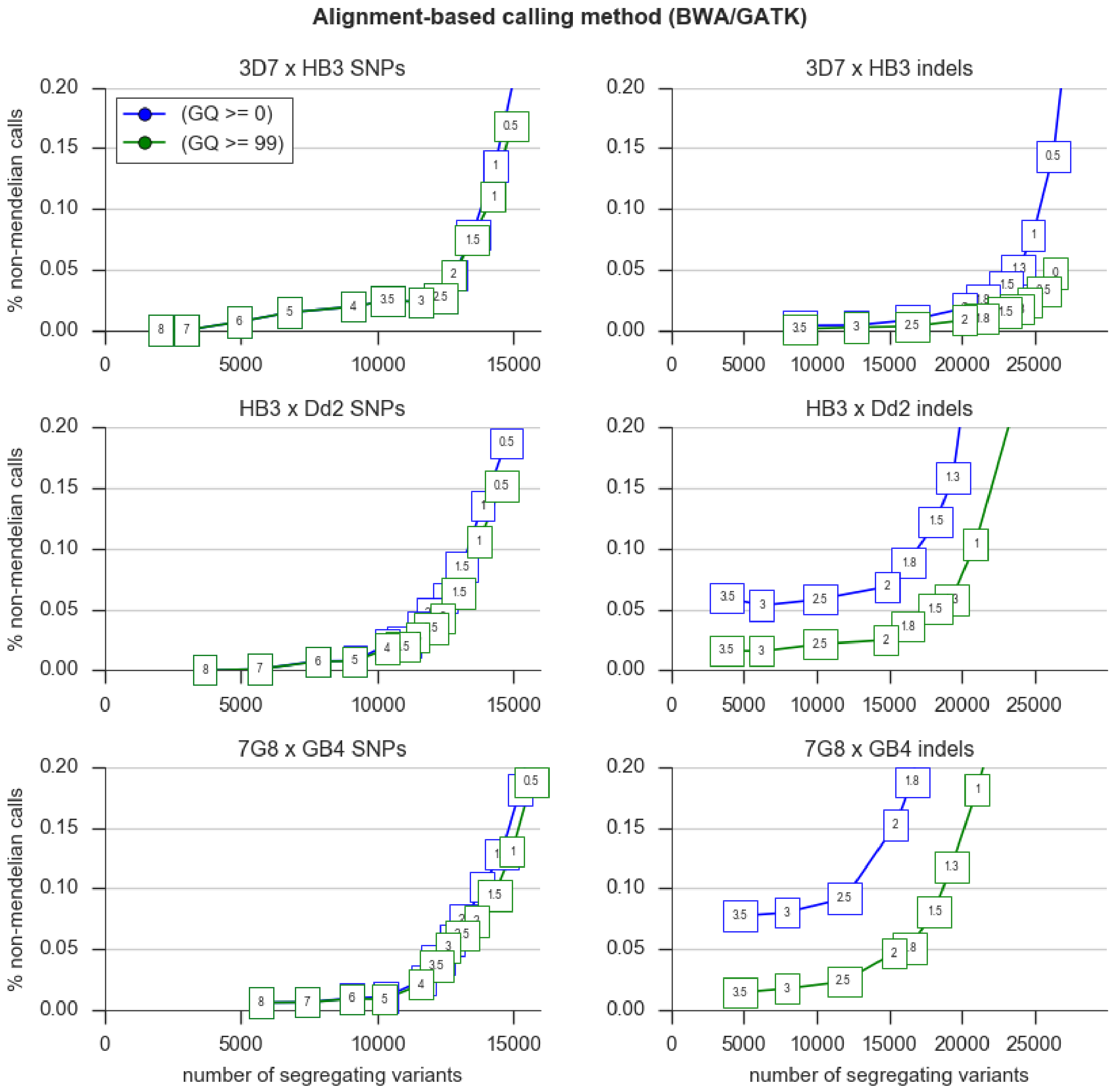

Figure S5*: Using Mendelian error as a guide to filtering variants and genotype calls from the assembly-based calling method. Each point plotted corresponds to variants filtered according to a minimum value of the SITE_CONF annotation and genotype calls filtered according to a minimum value of GT_CONF. The SITE_CONF threshold value is shown labelling the point, the colour indicates the GT_CONF threshold according to the legend.*

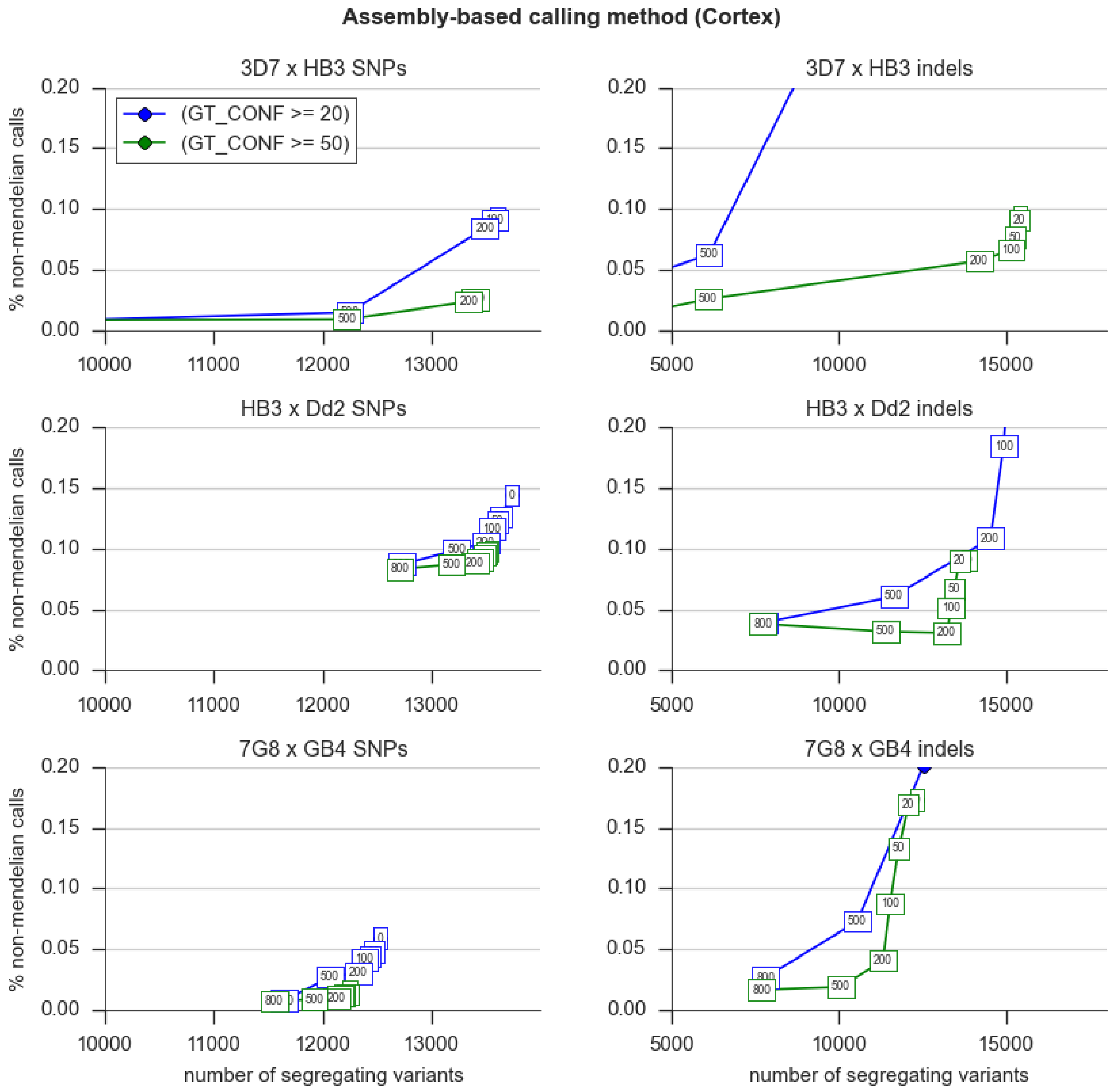

Figure S6: *Illustration of the alignment-based callset before and after variant filtration. The upper plot shows the raw variant calls, the lower plot shows the filtered variant calls. The main subplot in each shows each sample as a row and each variant as a column, painting genotype calls as follow: red: parent 1 allele; blue: parent 2 allele; white: missing genotype call; grey: filtered genotype call; yellow: parent genotype missing; black: non-Mendelian genotype; orange: reference allele and both parents reference also; green: alternate allele and both parents alternate also. Lines from the inheritance subplot to the accessibility track indicate the physical position of variants, with one line drawn for every 100 variants in the upper (unfiltered) plot and one line for every 10 variants in the lower (filtered) plot.*

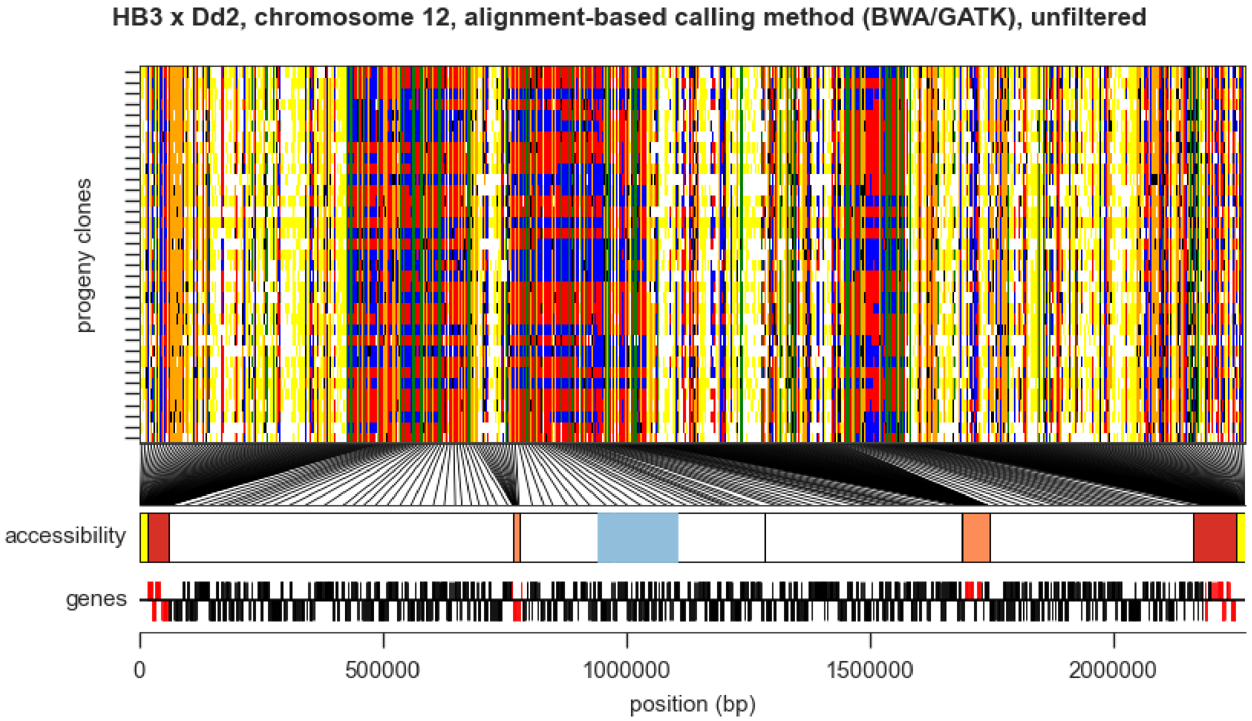

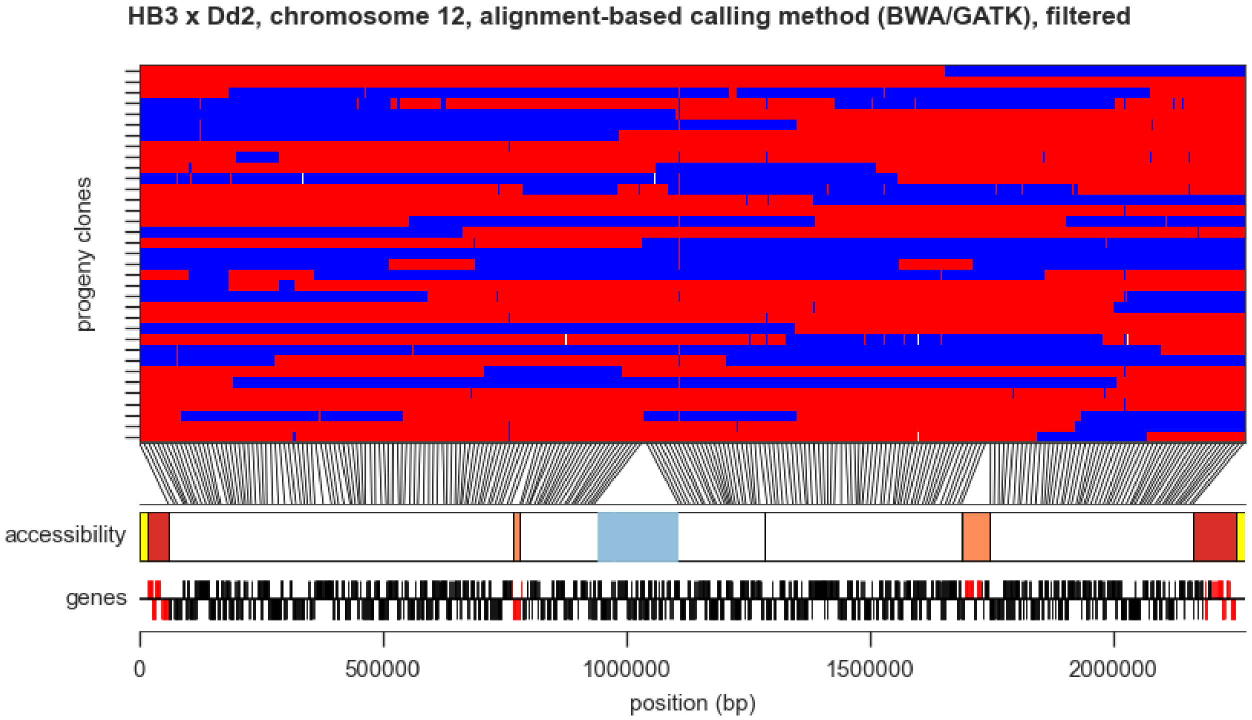

Figure S7: *Illustration of the assembly-based callset before and after variant filtration. The upper plot shows the raw variant calls, the lower plot shows the filtered variant calls. The main subplot in each shows each sample as a row and each variant as a column, painting genotype calls as described in Figure S7.*

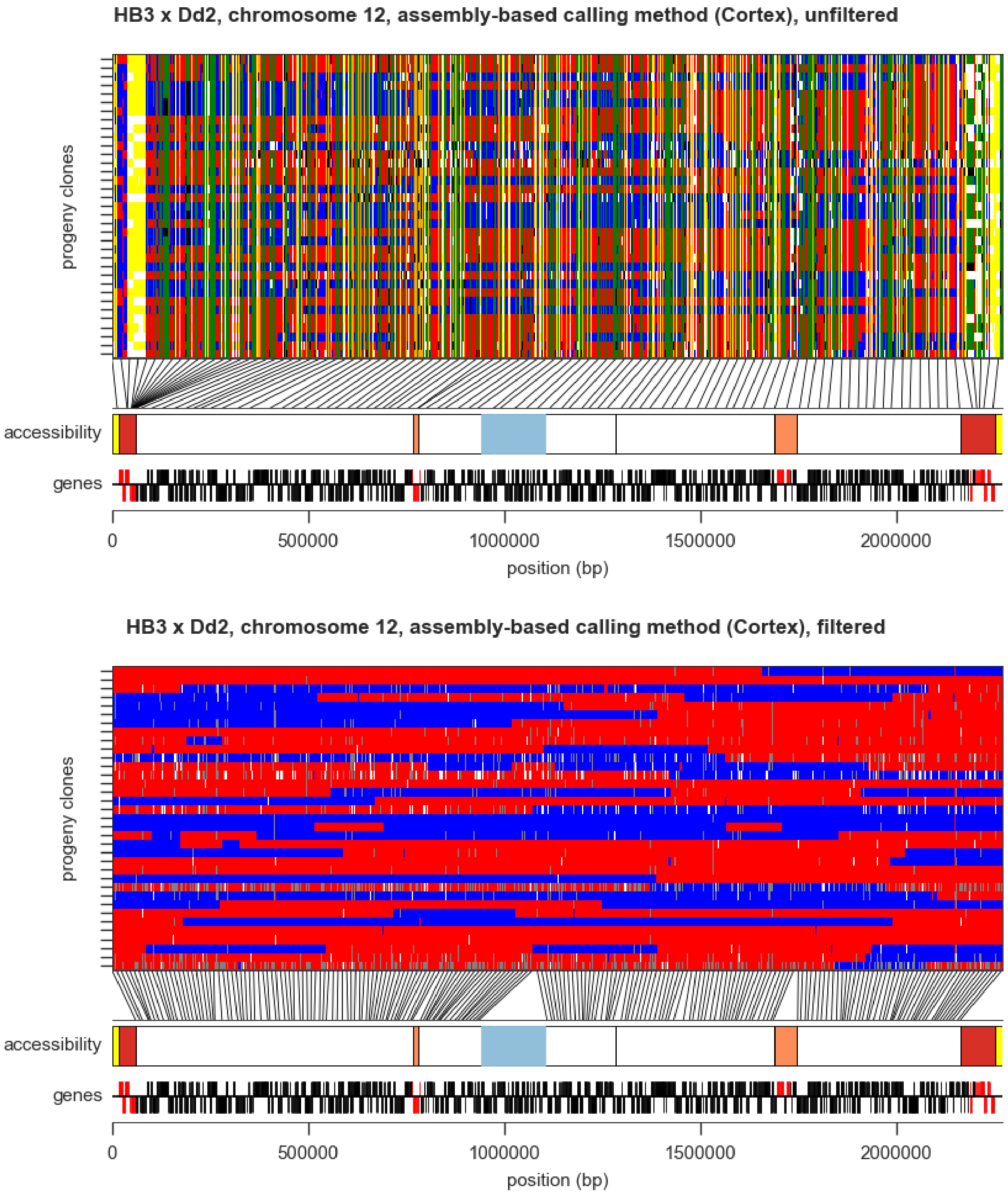

Figure S8*: Comparison of SNP and INDEL calls. The main subplot in each plot shows each sample as a row and each variant as a column, painting genotype calls as described in Figure S7. Lines from the inheritance subplot to the accessibility track indicate the physical position of variants, with one line drawn for every 10 variants.*

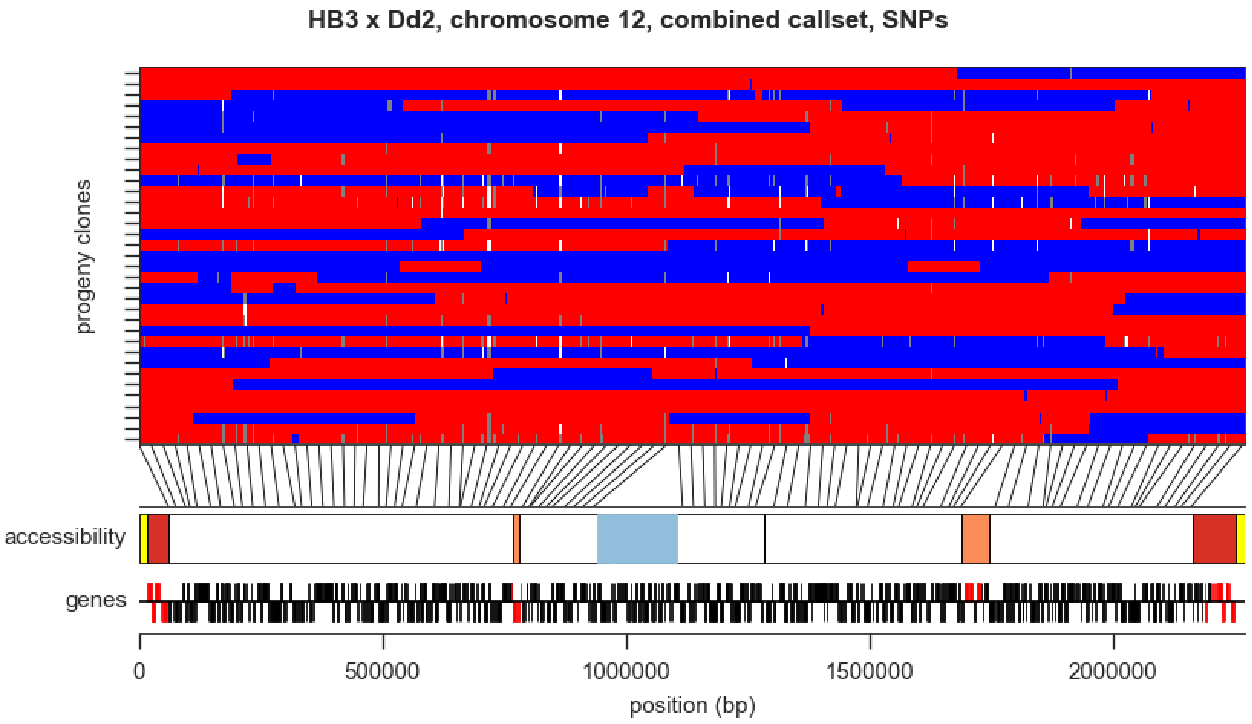

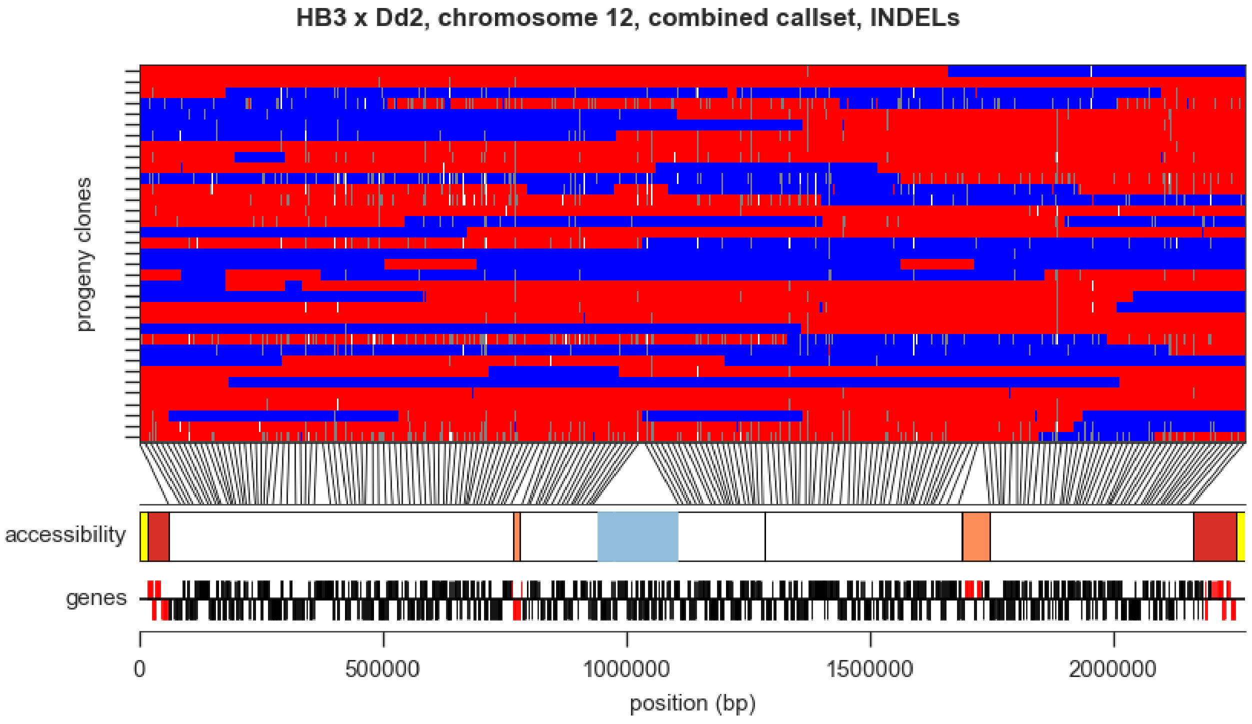

Figure S9*: Alignment and assembly at a locus where clone HB3 harbours a highly diverged gene sequence. A pileup of sequence reads is shown in blue for each of the parental clones, with mismatches coloured red, generated using LookSeq. Below each pileup is a representation of the reference (REF) and variant (ALT) contigs assembled by Cortex in regions of variation (each linked pair of REF and ALT contigs represents a bubble found in the assembly graph). For both replicates of clone HB3 alignment fails in large regions of gene msp1 however Cortex assembles contigs spanning these alignment gaps.*

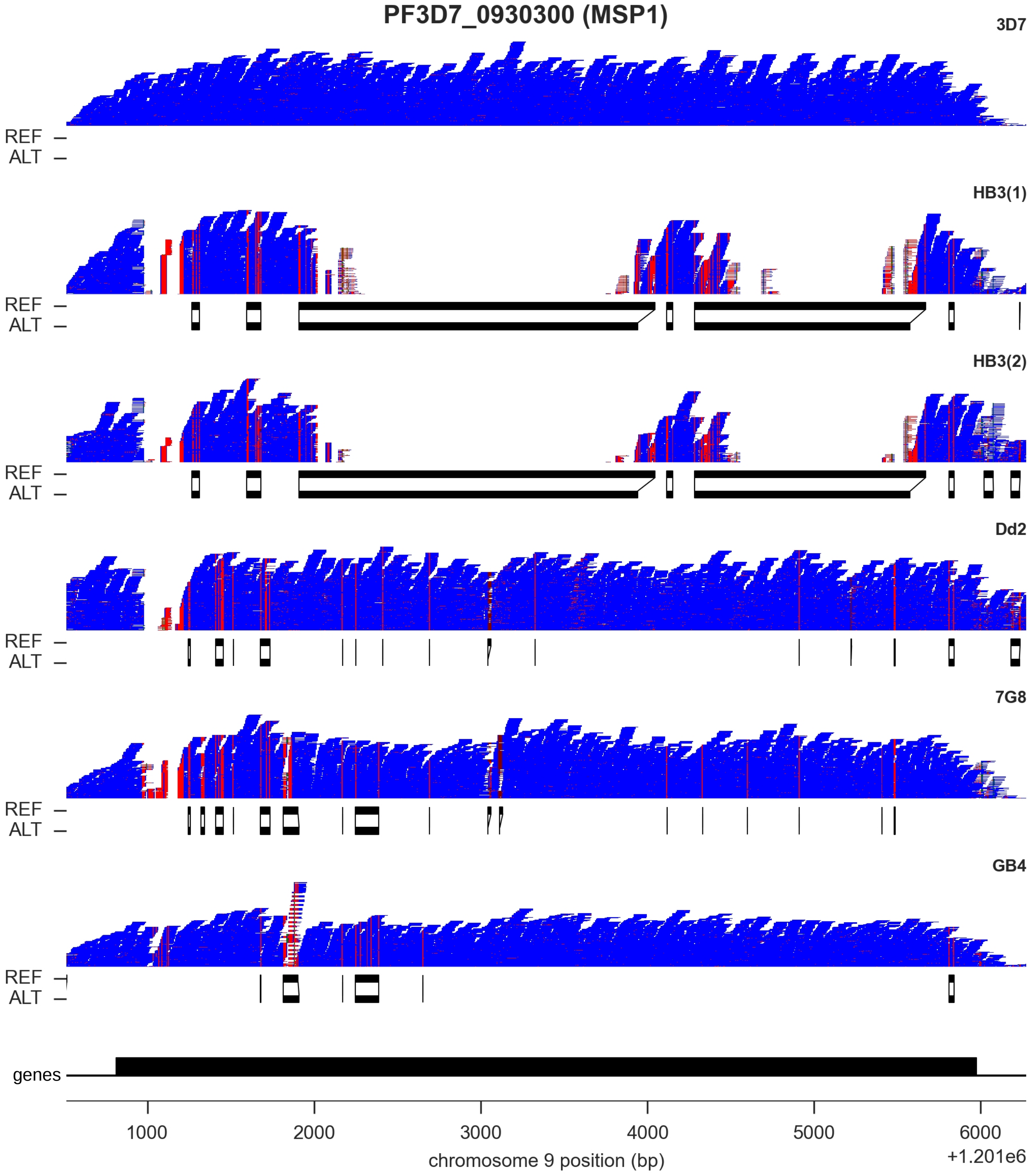

__Figure S10__: Size distribution for haplotype blocks transmitted from parents to progeny in the three crosses. "Parent 1" means the first named parent in each cross, i.e., 3D7 for the cross 3D7 x HB3.

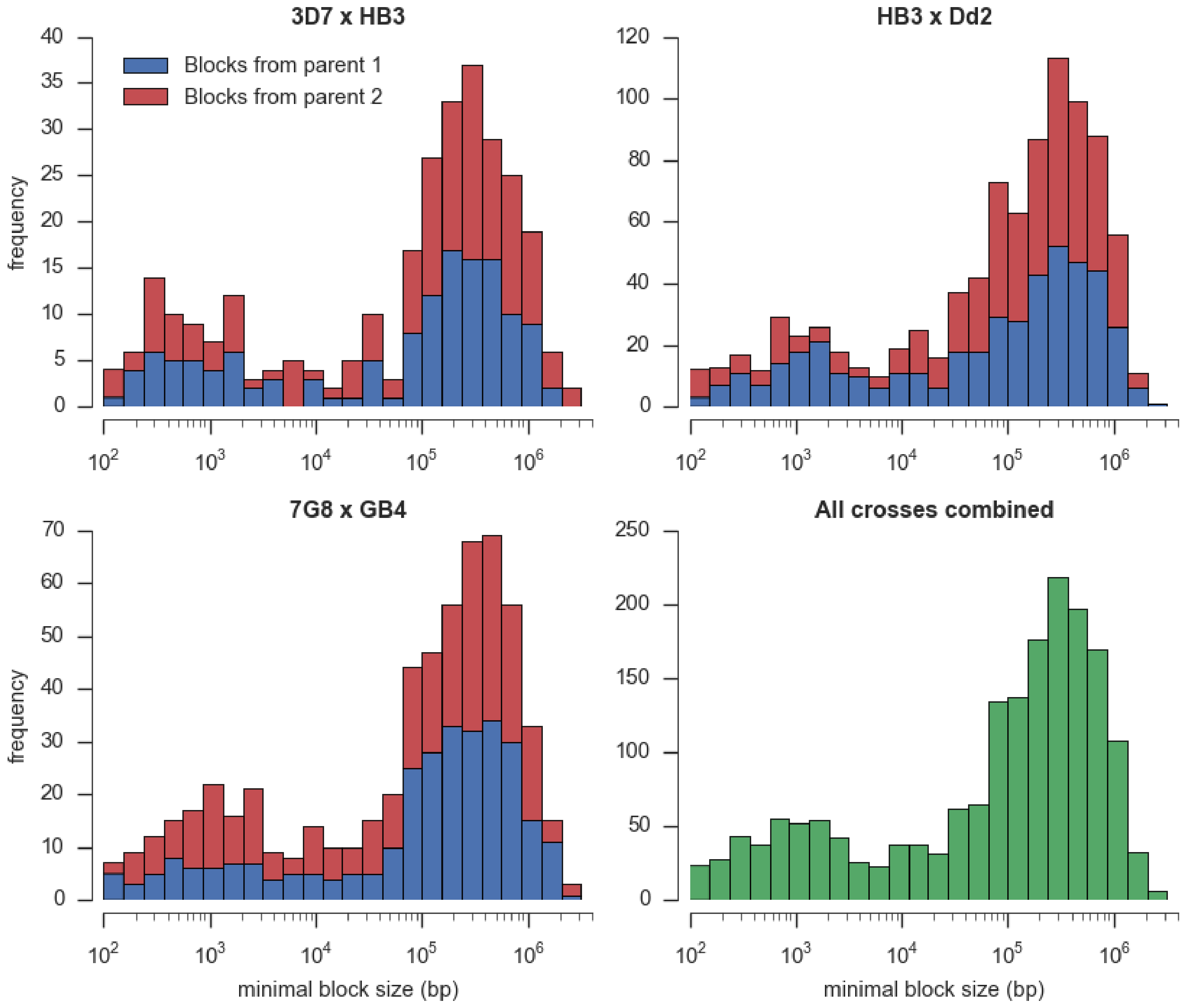

Figure S11: *Physical locations of CO and NCO recombination events observed. Events are shown for each of the 14 nuclear chromosomes. The lower track for each chromosome shows the genome region classification, with colours as in Figure S3.*

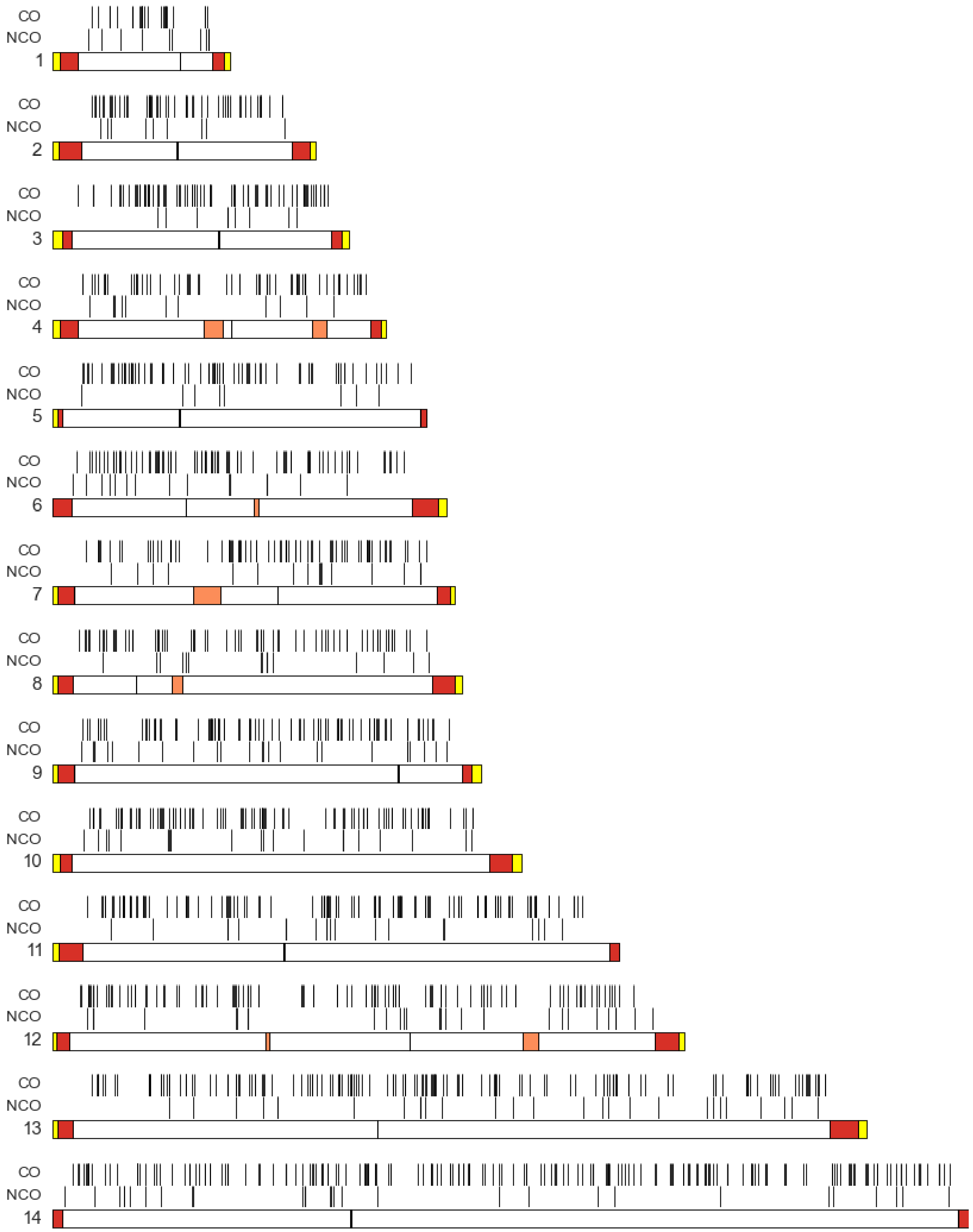

***Figure S12**: Long-range complex recombination events.*

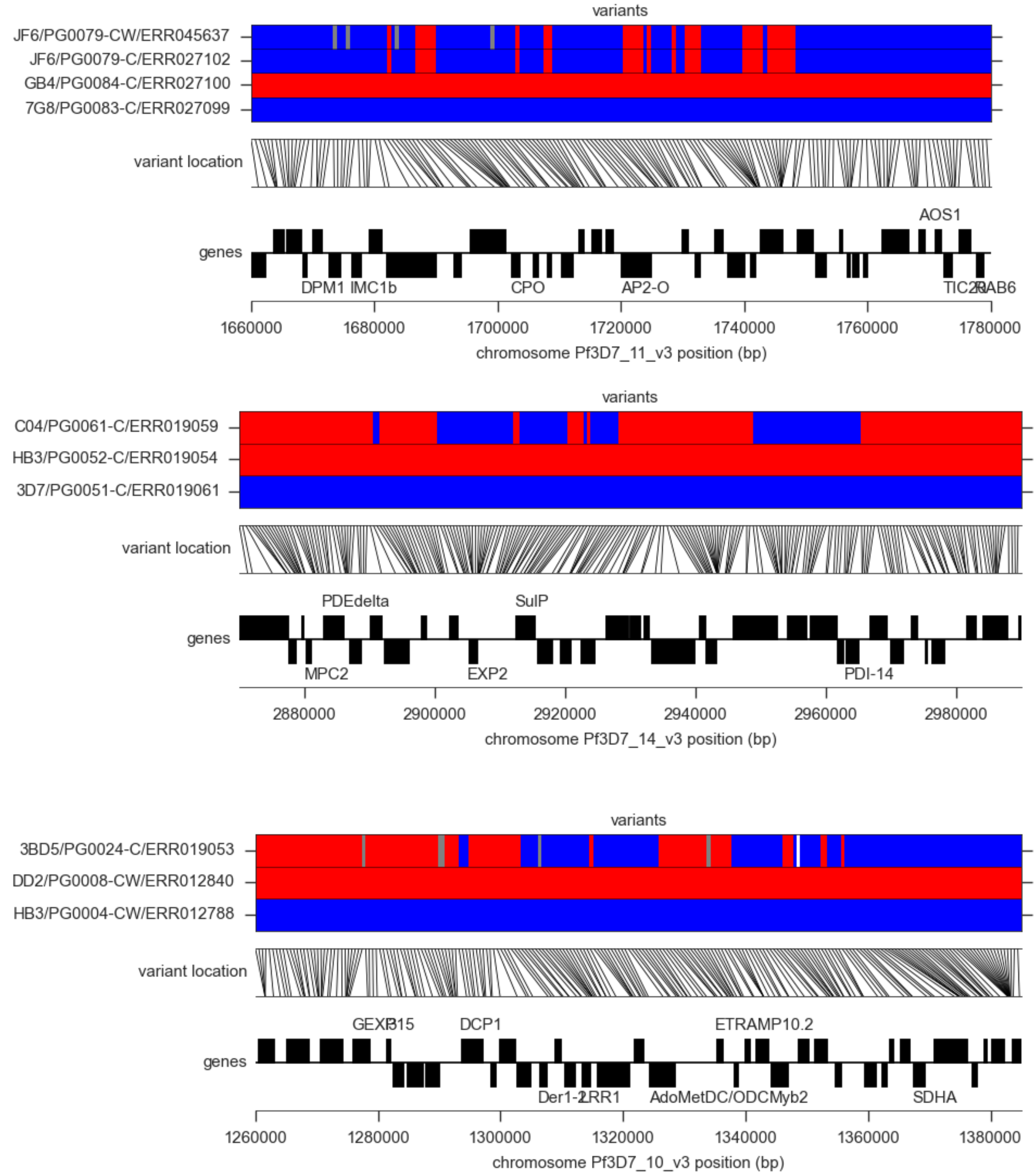

Figure S13: *CNV spanning MDR1 in the HB3xDd2 cross. A, evidence for CNV in the parent clones, as per Figure 4 in the main text. B, copy number prediction for all parents and progeny for chromosome 5.*

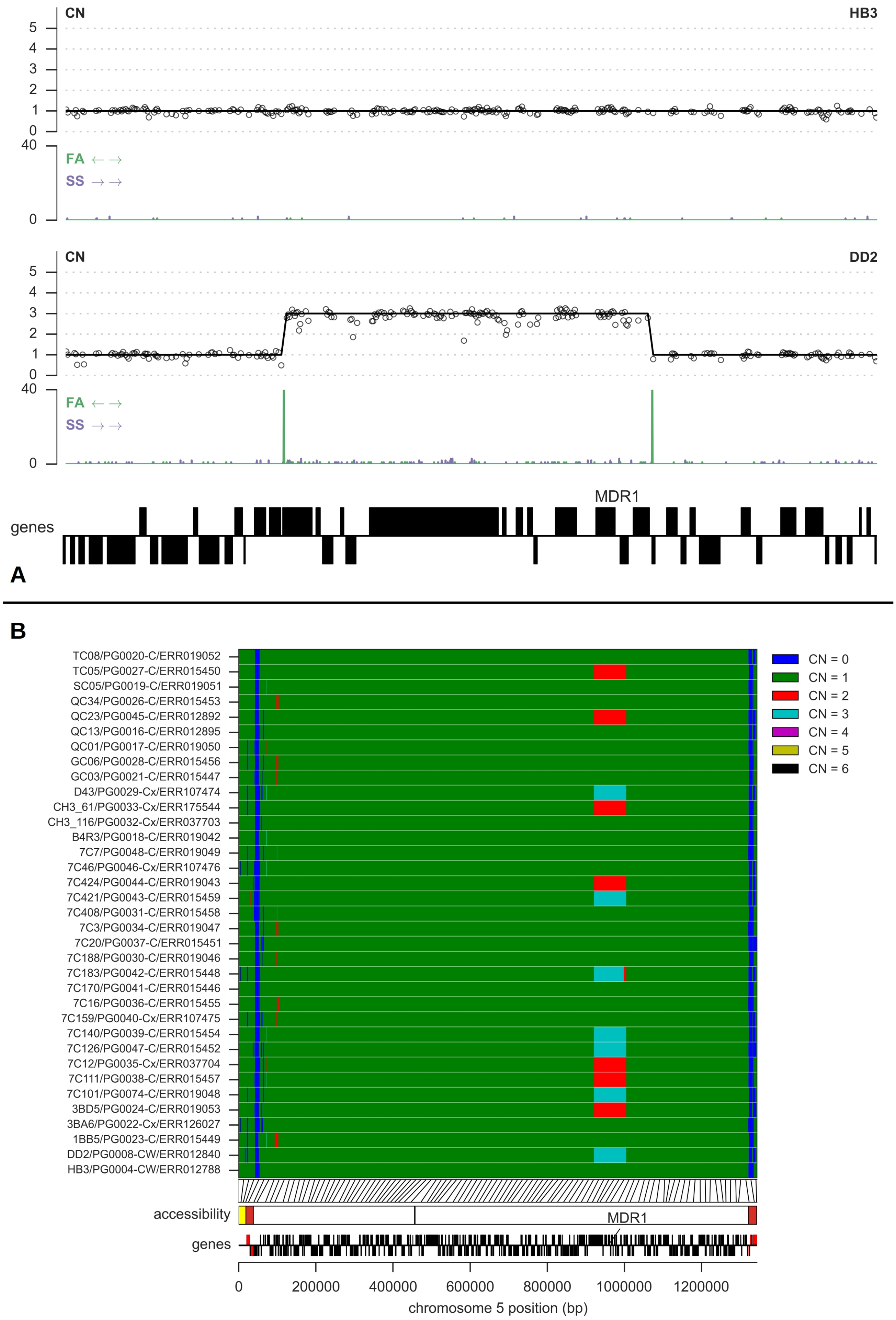

Figure S14: *Copy number prediction for all parents and progeny of cross 3D7xHB3 for chromosome 12 (including the gch1 locus).*

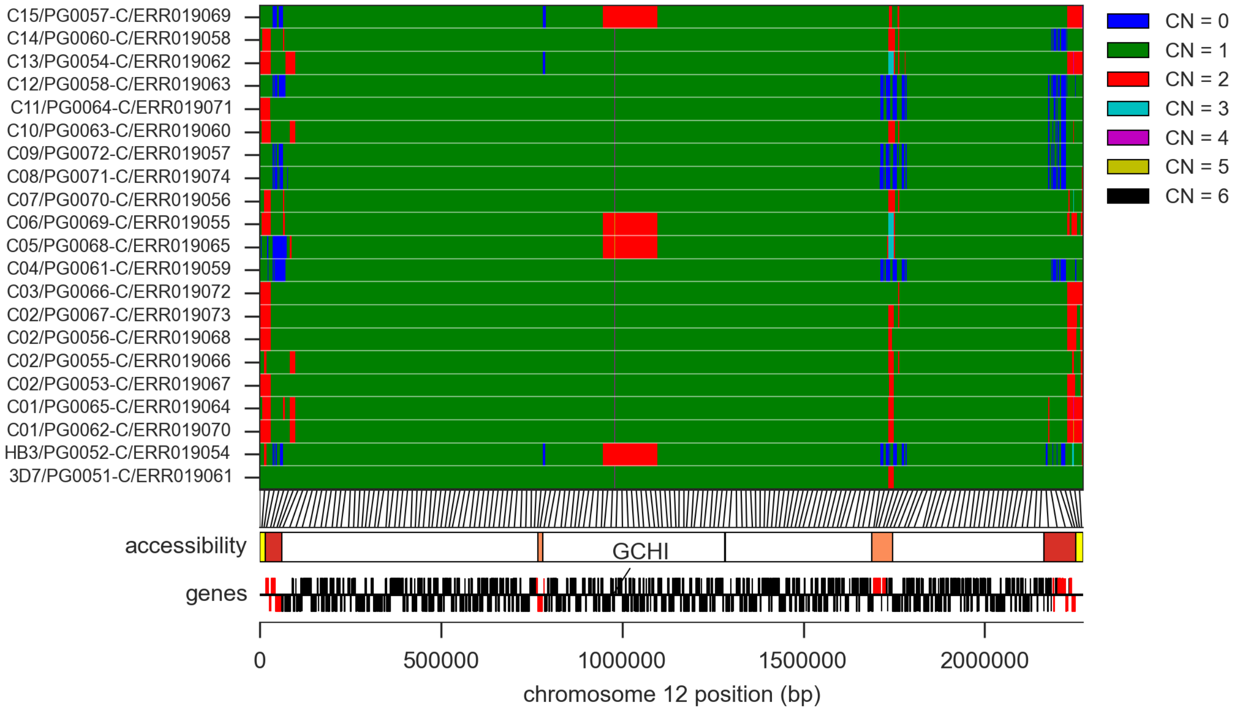

Figure S15: *Copy number prediction for all parents and progeny of cross HB3xDd2 for chromosome 12 (including the gch1 locus).*

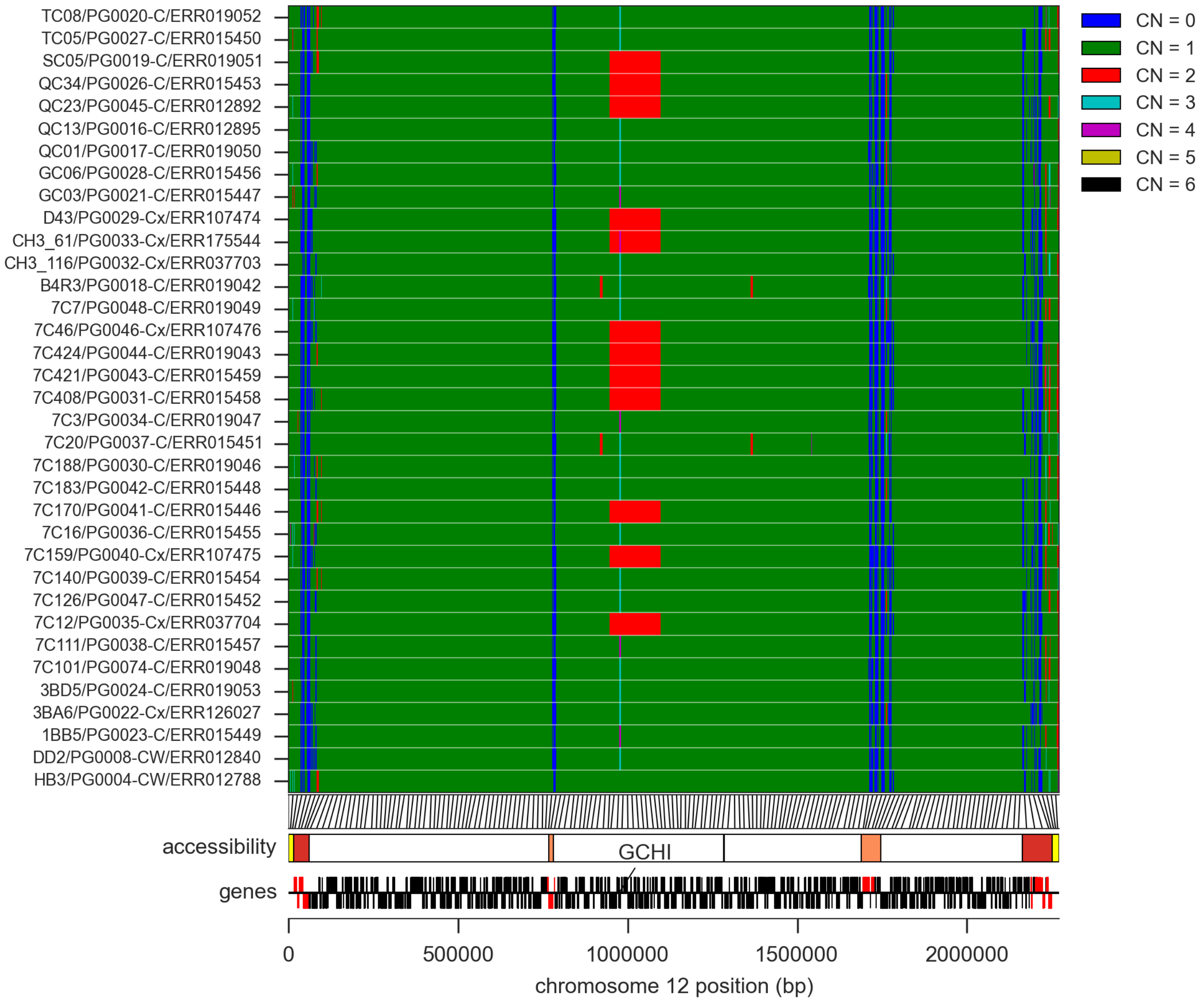

Figure S16*: Copy number prediction for all parents and progeny of cross 7G8xGB4 for chromosome 12 (including the gch1 locus).*

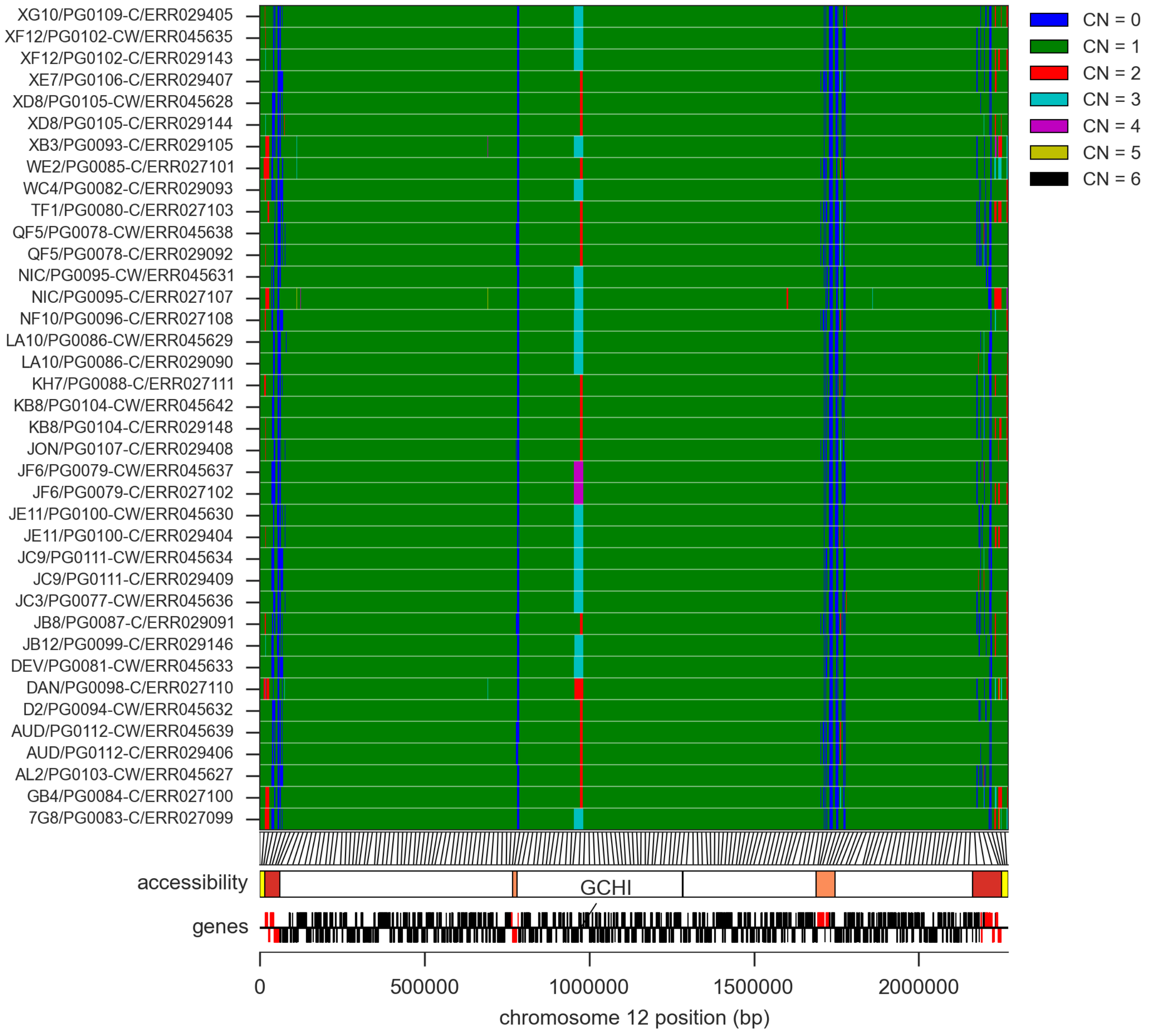

Figure S17: *Variant concordance between previously published sequences for clone HB3. Each Venn diagram shows the number of variants present in the HB3 sequences relative to the 3D7 reference genome.*

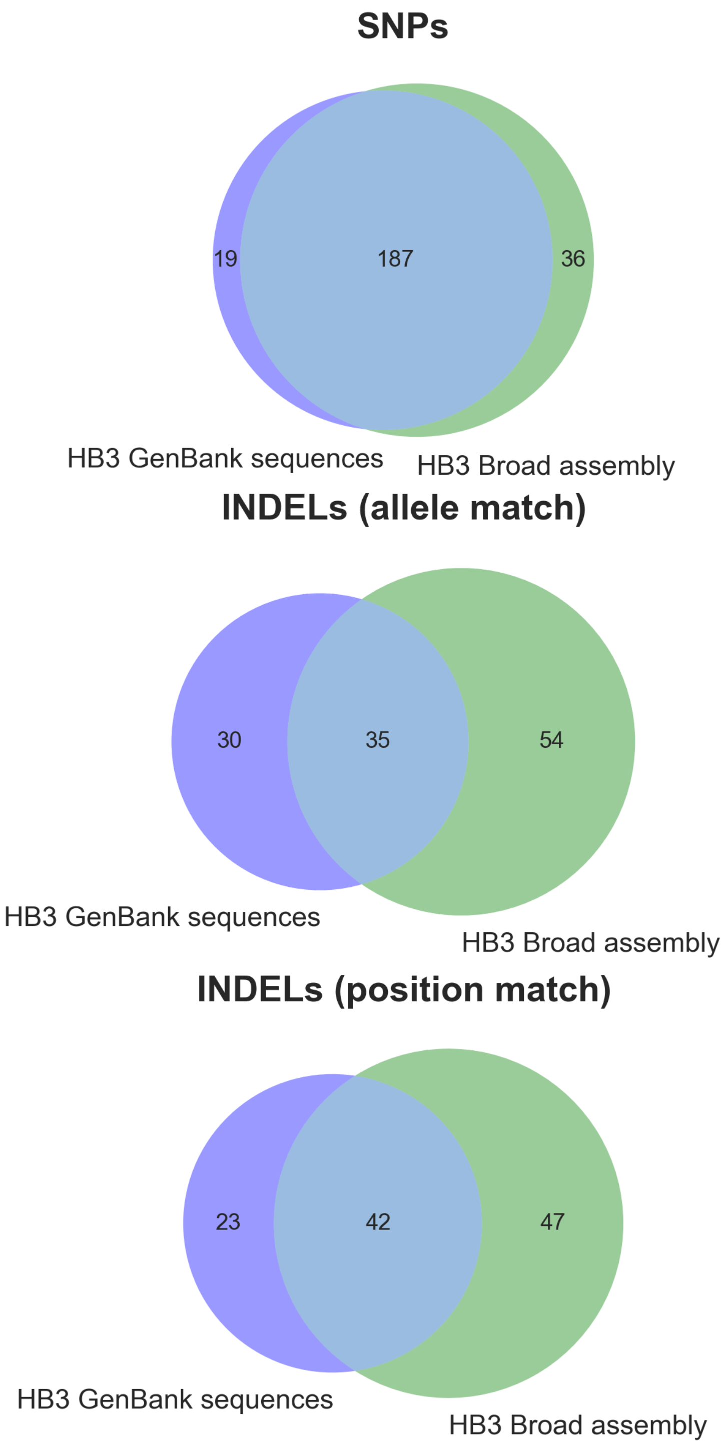

Figure S18. *INDEL polymorphism within tandem repeats by repeat unit length and tract length. Tandem repeat sequences were identified within the reference genome following (Montgomery et al. 2013). The fraction polymorphic is computed over all three crosses.*

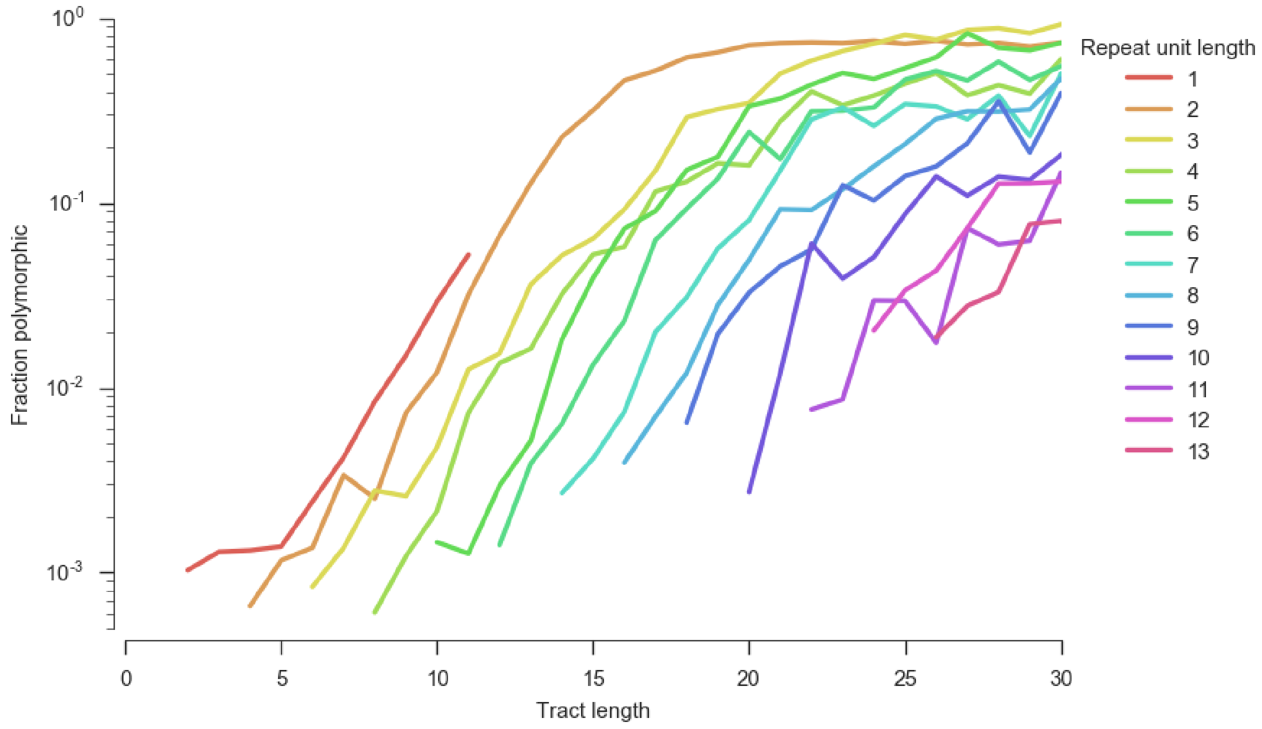

Figure S19. *Screenshots from the Web application at www.malariagen.net/apps/pf-crosses providing access to sequence and variation data on the three crosses.* ***A****, Introduction page, providing navigation to different tools for data exploration.* ***B****, Browse and query data on variants (SNPs and INDELs) discovered in the crosses by different calling methods.* ***C****, Browse genotype calls in parents and progeny and visualise patterns of allelic inheritance and recombination.* ***D****, Genome browser, providing multi-resolution views of various data tracks including coverage and mapping quality.* ***E****, Sequence alignment browser (LookSeq).*

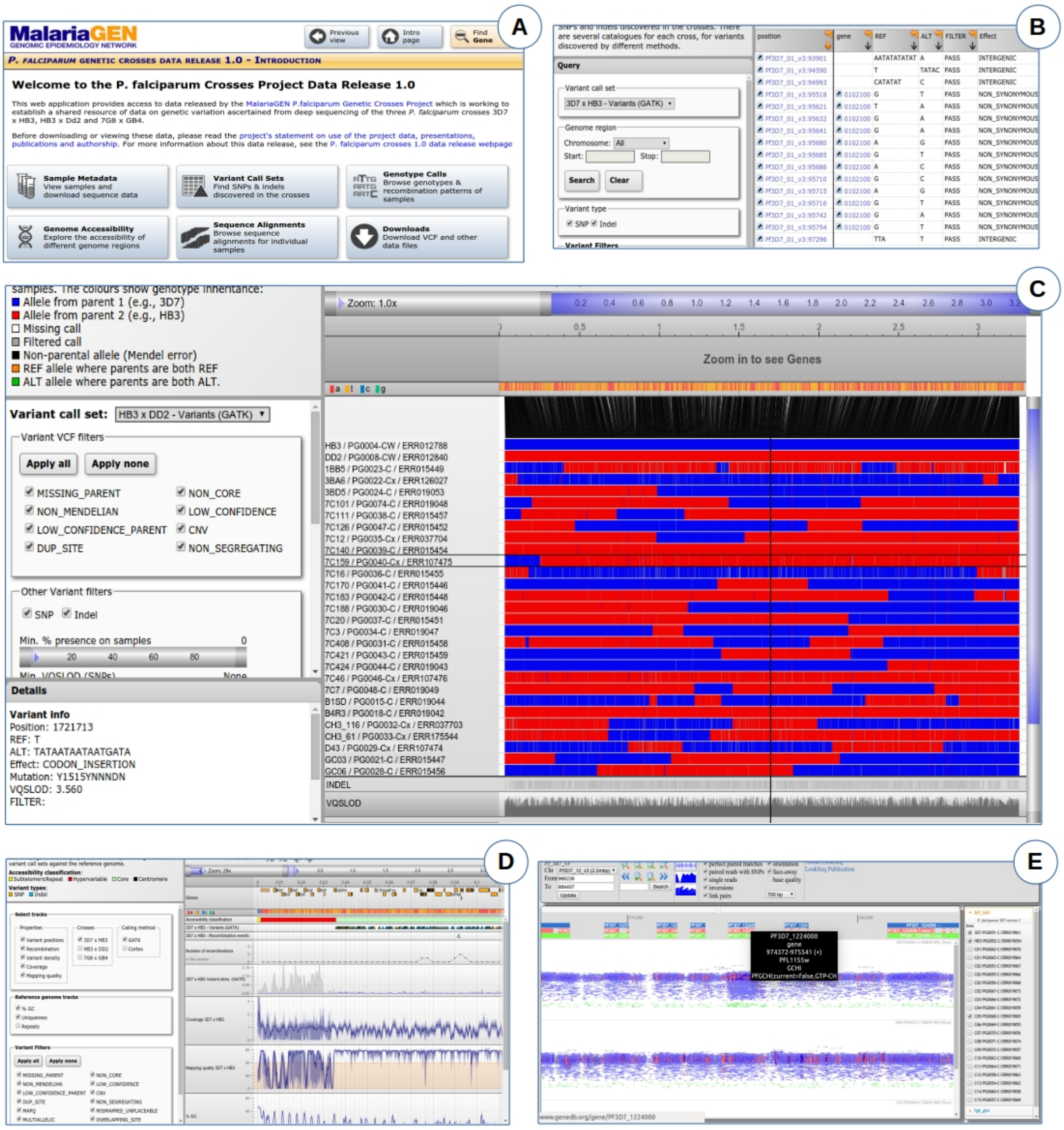

4. References

Abyzov A, Urban AE, Snyder M, Gerstein M. 2011. CNVnator: an approach to discover, genotype, and characterize typical and atypical CNVs from family and population genome sequencing. *Genome Res* **21**: 974–84. http://www.pubmedcentral.nih.gov/articlerender.fcgi?artid=3106330&tool=pmcentrez&rendertype=abstract (Accessed March 23, 2015).

Birren B, Lander E, Galagan J, Nusbaum C, Devon K, Henn M, Jaffe D, Butler J, Alvarez P, Gnerre S, et al. 2006. Plasmodium falciparum HB3, whole genome shotgun sequencing project. http://www.ncbi.nlm.nih.gov/nuccore/AANS00000000.

Chung WY, Gardiner DL, Anderson KA, Hyland CA, Kemp DJ, Trenholme KR. 2007. The CLAG/RhopH1 locus on chromosome 3 of Plasmodium falciparum: two genes or two alleles of the same gene? *Mol Biochem Parasitol* **151**: 229–32. http://www.ncbi.nlm.nih.gov/pubmed/17166605 (Accessed December 16, 2014).

DePristo MA, Banks E, Poplin R, Garimella K V, Maguire JR, Hartl C, Philippakis AA, del Angel G, Rivas MA, Hanna M, et al. 2011. A framework for variation discovery and genotyping using next-generation DNA sequencing data. *Nat Genet* **43**: 491–8. http://www.pubmedcentral.nih.gov/articlerender.fcgi?artid=3083463&tool=pmcentrez&rendertype=abstract (Accessed July 9, 2014).

Fidock DA, Nomura T, Talley AK, Cooper RA, Dzekunov SM, Ferdig MT, Ursos LM, Sidhu AB, Naudé B, Deitsch KW, et al. 2000. Mutations in the P. falciparum digestive vacuole transmembrane protein PfCRT and evidence for their role in chloroquine resistance. *Mol Cell* **6**: 861–71. http://www.pubmedcentral.nih.gov/articlerender.fcgi?artid=2944663&tool=pmcentrez&rendertype=abstract (Accessed February 11, 2015).

Hilliker AJ, Harauz G, Reaume AG, Gray M, Clark SH, Chovnick A. 1994. Meiotic gene conversion tract length distribution within the rosy locus of Drosophila melanogaster. *Genetics* **137**: 1019–26. http://www.pubmedcentral.nih.gov/articlerender.fcgi?artid=1206049&tool=pmcentrez&rendertype=abstract (Accessed December 16, 2014).

Hinterberg K, Mattei D, Wellems TE, Scherf A. 1994. Interchromosomal exchange of a large subtelomeric segment in a Plasmodium falciparum cross. *EMBO J* **13**: 4174–80. http://www.pubmedcentral.nih.gov/articlerender.fcgi?artid=395340&tool=pmcentrez&rendertype=abstract (Accessed November 11, 2014).

Iqbal Z, Turner I, McVean G. 2013. High-throughput microbial population genomics using the Cortex variation assembler. *Bioinformatics* **29**: 275–6. http://www.pubmedcentral.nih.gov/articlerender.fcgi?artid=3546798&tool=pmcentrez&rendertype=abstract (Accessed July 24, 2015).

Kozarewa I, Ning Z, Quail MA, Sanders MJ, Berriman M, Turner DJ. 2009. Amplification-free Illumina sequencing-library preparation facilitates improved mapping and assembly of (G+C)-biased genomes. *Nat Methods* **6**: 291–5. http://dx.doi.org/10.1038/nmeth.1311 (Accessed October 31, 2014).

Li H, Durbin R. 2009. Fast and accurate short read alignment with Burrows-Wheeler transform. *Bioinformatics* **25**: 1754–60. http://www.pubmedcentral.nih.gov/articlerender.fcgi?artid=2705234&tool=pmcentrez&rendertype=abstract (Accessed July 9, 2014).

Manske HM, Kwiatkowski DP. 2009. LookSeq: a browser-based viewer for deep sequencing data. *Genome Res* **19**: 2125–32. http://www.pubmedcentral.nih.gov/articlerender.fcgi?artid=2775587&tool=pmcentrez&rendertype=abstract (Accessed December 5, 2014).

Manske M, Miotto O, Campino S, Auburn S, Almagro-Garcia J, Maslen G, O'Brien J, Djimde A, Doumbo O, Zongo I, et al. 2012. Analysis of Plasmodium falciparum diversity in natural infections by deep sequencing. *Nature* **487**: 375–9. http://www.pubmedcentral.nih.gov/articlerender.fcgi?artid=3738909&tool=pmcentrez&rendertype=abstract (Accessed November 11, 2014).

McKenna A, Hanna M, Banks E, Sivachenko A, Cibulskis K, Kernytsky A, Garimella K, Altshuler D, Gabriel S, Daly M, et al. 2010. The Genome Analysis Toolkit: a MapReduce framework for analyzing next-generation DNA sequencing data. *Genome Res* **20**: 1297–303. http://www.pubmedcentral.nih.gov/articlerender.fcgi?artid=2928508&tool=pmcentrez&rendertype=abstract (Accessed July 9, 2014).

Montgomery SB, Goode DL, Kvikstad E, Albers CA, Zhang ZD, Mu XJ, Ananda G, Howie B, Karczewski KJ, Smith KS, et al. 2013. The origin, evolution, and functional impact of short insertion-deletion variants identified in 179 human genomes. *Genome Res* **23**: 749–61. http://www.pubmedcentral.nih.gov/articlerender.fcgi?artid=3638132&tool=pmcentrez&rendertype=abstract (Accessed August 21, 2014).

Ranford-Cartwright LC, Mwangi JM. 2012. Analysis of malaria parasite phenotypes using experimental genetic crosses of Plasmodium falciparum. *Int J Parasitol* **42**: 529–34. http://www.pubmedcentral.nih.gov/articlerender.fcgi?artid=4039998&tool=pmcentrez&rendertype=abstract (Accessed November 15, 2014).

Samarakoon U, Regier A, Tan A, Desany BA, Collins B, Tan JC, Emrich SJ, Ferdig MT. 2011. High-throughput 454 resequencing for allele discovery and recombination mapping in Plasmodium falciparum. *BMC Genomics* **12**: 116. http://www.pubmedcentral.nih.gov/articlerender.fcgi?artid=3055840&tool=pmcentrez&rendertype=abstract (Accessed November 11, 2014).

Sepúlveda N, Campino SG, Assefa SA, Sutherland CJ, Pain A, Clark TG. 2013. A Poisson hierarchical modelling approach to detecting copy number variation in sequence coverage data. *BMC Genomics* **14**: 128. http://www.pubmedcentral.nih.gov/articlerender.fcgi?artid=3679970&tool=pmcentrez&rendertype=abstract (Accessed November 11, 2014).

Van der Auwera GA, Carneiro MO, Hartl C, Poplin R, Del Angel G, Levy-Moonshine A, Jordan T, Shakir K, Roazen D, Thibault J, et al. 2013. *Current Protocols in Bioinformatics*. eds. A. Bateman, W.R. Pearson, L.D. Stein, G.D. Stormo, and J.R. Yates. John Wiley & Sons, Inc., Hoboken, NJ, USA http://www.pubmedcentral.nih.gov/articlerender.fcgi?artid=4243306&tool=pmcentrez&rendertype=abstract (Accessed July 16, 2014).

Youds JL, Boulton SJ. 2011. The choice in meiosis - defining the factors that influence crossover or

non-crossover formation. *J Cell Sci* **124**: 501–13.
http://www.ncbi.nlm.nih.gov/pubmed/21282472 (Accessed December 12, 2014).
